# The Gendered Toy Choice (GTC): validating a behavioral measure of gendered parenting

**DOI:** 10.3389/fpsyg.2025.1601339

**Published:** 2025-07-29

**Authors:** Noya Kislev, Tamar Saguy

**Affiliations:** Baruch Ivcher School of Psychology, Reichman University, Herzliya, Israel

**Keywords:** gendered parenting, measurement, validity, Gendered Toy Choice measure, gendered behavior

## Abstract

*Gendered parenting* reflects parents' tendency to promote gender-typed behaviors of their children, shaping their everyday experiences and environments. Existing research primarily relies on self-report or observational methods, limiting behavioral insights. This study introduces the development and validation of the Gender Toy Choice (GTC) measure, an unobtrusive behavioral tool assessing parents' real-time product choices for their children. In three studies conducted among Israeli and U.S. parents to preschool children, the GTC measure demonstrated face, construct, concurrent, convergent, and discriminant validity, supporting its theoretical relevance. Findings highlight how parental choices reinforce gender norms in children's daily lives and provide a standardized behavioral measure for future research. By offering a novel, easy-to-implement tool, this work contributes to the study of factors underlying parents' gendered decision-making and the mechanisms shaping gendered parenting practices.

## Introduction

An individual is considered gender-typed when he or she thinks and behaves in ways that are consistent with gender stereotypes (Leaper and Bigler, 2018); and gender-typing is the process through which such views and behaviors are acquired and internalized (Turner and Gervai, 1995). Various factors contribute to children's gender-typing, including those rooted in biological predispositions, in cognitive-developmental processes, and in environmental pressures (Endendijk et al., 2018). Our focus is on the parental influence on this process, which is especially pertinent in young children's lives. This influence is often referred to as gendered parenting, which is parents' tendency to promote the gender-typing of their young children (Morawska, 2020).

Gender typing, or the adherence to gender stereotypes in early years, is often considered to be a learned process. Much research in this domain was driven by the theoretical frameworks of social learning theory (Bussey and Bandura, 1999), gender schema theory (Bem, 1981; Martin and Halverson, 1981), and associated cognitive-development theories (Bigler and Liben, 2007; see also Arthur et al., 2008), which all share the notion that young children are highly attuned to the expectations of others regarding how girls and boys should and should not behave (or be), and use this information to further act on their environment in terms of choices, and processing of information (Bandura, 1965).

Thus, research on gendered parenting considered a range of ways via which children can pick up on parents' gendered expectations (Blakemore et al., 2009; Ruble et al., 2007). This work underscores influences related to modeling, explicit and implicit communication of gender-typed expectations, and the construction of children's environments (e.g., with gender-typed toys; Leaper and Bigler, 2018; Morawska, 2020). Via these different channels, parents communicate messages that convey information, either explicitly or implicitly, about how girls and boys are supposed (and are not supposed) to think, feel or behave. It includes, on one hand, practices that encourage and promote children's engagement in gender-stereotypic domains (such as dancing for girls and socker for boys) and reinforcement of behaviors that are consistent with gender stereotypes (e.g., certain forms of play, emotional expressions, etc.); and on the other hand, limitation or discouragement of counter-stereotypic experiences, and even sanctioning a non-conforming gender behavior (Mesman and Groeneveld, 2017).

Gendered parenting can potentially have long-term effects, as research indicates that young children are sensitive to their parents' gendered preferences. For instance, preschoolers were shown to anticipate their parents will have gender-typed expectations regarding toy preferences (Freeman, 2007). Longitudinal research further suggests that these gendered expectations can influence children's educational trajectories and shape their future pursuits and life choices, often reinforcing traditional gender norms and stereotypes (e.g., Martin et al., 2002). Consequently, gendered parenting practices may lead children to engage in activities aligned with traditional gender roles while avoiding those that do not conform to these stereotypes, thereby perpetuating the existing gender status quo (Kane, 2009; Halpern and Perry-Jenkins, 2016; Leaper and Bigler, 2018). However, existing measures of gendered parenting largely rely on self-reports, which may be susceptible to social desirability bias and limited introspective access. There is a need for behavioral tools that capture implicit or less conscious parental tendencies that affect the way they shape their children's environment.

The goal of the current work is to develop a behavioral measure centered on toy choices made by parents for their children. This focus reflects the profound role of play in early childhood development and the significant influence of toys—and more broadly, parents' design of their children's environment—as a medium for shaping developmental pathways (e.g., Klemenović, 2014; Kultti and Samuelsson, 2017). As elaborated below, previous research has highlighted the role of toys in reinforcing or challenging gender norms, yet this work did not involve behavioral assessment. The goal of the current work was to propose a behavioral, valid and scalable measure of this aspect of gendered parenting—choice of toys, that advances this line of inquiry.

### How is gendered parenting assessed

Notwithstanding the importance of existing research on gendered parenting, most of it involved observational or self-report assessments. Some previous work has also utilized toys as a basis for measuring gendered parenting, however none of this research was behavioral. We will describe the measures used in previous research and their shortcomings, which our new measure is intended to address.

### Use of language as a measure of gendered parenting

It was found that parents tend to use gendered language (Gelman et al., 2004), and talk differently with their daughters vs. their sons (Leaper et al., 1998; Aznar and Tenenbaum, 2015). A meta-analysis on parent's use of language with their children (Leaper et al., 1998) showed that mothers tended to talk more, use more supportive and negative speech, and use less directive and informing speech than fathers did. In addition, mothers tended to talk more and use more supportive speech with daughters than with sons.

Similarly, Gelman (2004), examined language use during mother-child interaction, using a task involving the mother reading a book with mostly pictures to her child. The book depicted male and female figures engaging in activity that is either gender stereotypic or not. Most of the interaction between the mother and the child was spontaneous conversation generated by the books, and the session was videotaped. A content analysis of the conversations revealed that mothers expressed gender concepts mostly through implicit means, including reference to categories of gender, labeling of gender, and contrasting males vs. females. The authors concluded that there is much essentialist content in mother-child conversations, even for mothers who express gender egalitarian beliefs, and that mothers' linguistic input conveys subtle messages about gender from which children may construct their own essentialist beliefs (e.g., “girls like dolls”; Gelman, 2004). Another study that used a picture book to elicit conversation between parents and their children (Endendijk et al., 2014), found that fathers made more comments confirming gender stereotypes than mothers. Fathers with two boys were especially prone to emphasize appropriate male behavior in their gender talk (Endendijk et al., 2014).

Aznar and Tenenbaum (2015), also used story telling tasks, while parent-child interactions took place and were videotaped at home. The tasks involved generating stories about parents and their children and discussing them (e.g., “a time that the child fell”). The conversations' transcripts were coded for amount of talk and use of emotion words. Results showed that overall, mothers used a higher proportion of emotion words than did fathers, and that (at the age of 4), parents of daughters used more emotional language than did parents to sons (Aznar and Tenenbaum, 2015).

### Interactive behavior as a measure of gendered parenting

Other measures of gendered parenting focused on observation of play behavior of parents with their children (Mesman and Groeneveld, 2017). For example, in a free play situation, Bradley and Gobbart (1989) gave parents a selection of masculine (e.g., a hammer), feminine (e.g., a doll), and neutral (e.g., a cloth turtle) toys and recorded the first three toys the parents presented to their children. Parents also completed a scale that measured their gender role orientations. It was found that fathers with traditional gender role views offered more gender-typed toys than non-gender-typed toys, whereas mothers did not discriminate in their toy selection.

In another study (Fagot and Hagan, 1991), families were observed at home while playing. The family members were requested to interact as normally as they could, and these interactions were coded for type of activity and toy engaged with (masculine or feminine), form of interaction (attempts to communicate, aggressive behavior, etc.), reactions of parents in terms of style (directive, instructional activity, initiation), and valence (e.g., favorable comments, encouragement; criticism, verbal punishment, physical restraint, aggression). Most analysis did not reveal sex differences, however at the age of 18 months, it was found that fathers gave fewer positive reactions to boys engaging in female-typical toy play (Fagot and Hagan, 1991). Similarly, in studies in which parents and children were given toys to play in laboratory settings, parents showed more enthusiasm and involvement when presented with gender-typed toys for their child rather than cross-gender toys and spend more time playing with these toys, especially with their sons (Caldera and Sciaraffa, 1998; Wood et al., 2002).

While observational studies involving both language and interactions allow for in-depth analysis and understanding of communication patterns conveying implicit gendered messages from parents to child, they have notable drawbacks. First, it is expensive and complicated to enact an interactive study, given the amount of time and efforts that families are required to invest in order to participate, as well as the need to use complex coding systems, that requires much training. In addition, the mere presence of observants and the artificial setting and context of play, may affect parents and bias their behavior toward a more socially desired, gender egalitarian conduct. Indeed, when asked explicitly, parents tended to report egalitarian attitudes toward gender roles; However, when asked implicitly about the desirability of different toys for their own child, parents preferred gender-typed toys for their children (Kollmayer et al., 2018).

The complexity and cost of running such studies also renders them difficult to scale in the sense that it is extremely challenging to obtain large samples. Moreover, it renders the task of replicability extremely limited. One way to deal with such drawbacks is to have parents imagine they are interacting with their child and having them report on their behavioral intentions. For example, parents were asked how comfortable they would be with gender non-conforming behavior of their child (e.g., dressing up as the other sex), and how often they would do or say something to try to change this behavior (Spivey et al., 2018). Parents of boys reported significantly more discomfort with their child's engaging in gender-non-conforming behaviors and more frequent efforts to change these behaviors compared to parents of girls. In addition, egalitarian attitudes toward gender were associated with lower levels of discomfort with gender-non-conforming behaviors and fewer parent's efforts to change this behavior (Spivey et al., 2018). Such a measure obviously overcomes the problems of scalability but has a major deficiency of not assessing actual behavior.

### Intentions regarding purchasing toys

Other line of studies utilized the mere selection of toys, or reports about purchasing intentions (rather than context of interactive play) to assess gendered parenting. Weisgram and Bruun (2018), asked participants to report about the extent to which they are likely to buy different toys (varying in their gender-typicality) to their prospective children (“If you were to have a child in the future, please rate how likely you would be to buy these toys for your daughter”—followed by the identical question about a prospective son). In addition, the same participants were asked about their gender stereotypes of toys (“Who should play with each toy?”, with response options of “Only Boys”, “Only Girls”, or “Both Boys and Girls”). Prospective parents (both men and women) reported intentions to purchase gender-typed toys for their unborn children (i.e., plan to purchase masculine toys for prospective sons and feminine toys for prospective daughters), though both men and women also endorsed more stereotypes about feminine than masculine toys (Weisgram and Bruun, 2018).

Blakemore and Centers (2005) used a large American sample to rate 126 toys as suitable for boys, girls, or both. From these ratings, they established five categories of toys: strongly masculine, moderately masculine, neutral, moderately feminine, and strongly feminine. Using these categories, they constructed toy sets, each consisting of few toys from each category. Then (in their second study, using a different sample), they asked the participants to rate the toys on scales that measured their characteristics (e.g., to what extent does the toy encourage creativity, social play, is artistic, scientific, educational, exciting, encourages competition, develops physical skills, etc.). It was found that girls' toys were associated with physical attractiveness, nurturance, and domestic skill, whereas boys' toys were rated as more violent, competitive, exciting, and somewhat dangerous. We relied on Blakemore and Centers' (2005) work and findings in the development of the measure proposed in the current work.

As far as we are aware of, only a single study assessed the actual choice of toys on part of adults. Fisher-Thompson (1993) interviewed buyers at a toy-store and asked them which toys they have just purchased and for whom. Overall, customers were more likely to purchase gender-typed than non-gender-typed toys, especially when purchasing for a boy. This work, apart from being conducted over 30 years ago, did not focus on parenting, and suffers from similar drawbacks to observational studies, requiring much time and effort on part of researchers and observants.

Based on the literature reviewed above, it is clear that parental preferences and choices regarding children's toys are an important aspect of, and can be used to assess gendered parenting. Play during early childhood is widely recognized as a critical component of children's development. It contributes to multiple domains including cognitive, emotional, physical, and social growth. Through play, children explore their environment, express emotions, develop language and social skills, and engage in creative and imaginative thinking (Ginsburg, 2007; Lillard et al., 2013). Importantly, toys are not neutral objects—they often carry gendered meanings and reflect cultural expectations, thus serving as tools through which children internalize social roles and norms (Blakemore and Centers, 2005; Dinella and Weisgram, 2018). Because play is both a form of expression and a mode of learning, the toys children are encouraged to play with can influence their interests, abilities, and sense of identity. Parents play a central role in shaping children's play environments and guiding toy selection, making parental choices around toys a meaningful behavioral expression of gendered parenting.

Having said that, conducting observational and interaction studies to explore parental choices and behaviors regarding their children's toys involves great complexity and costs and is subjected to potential biases and threat to ecological validity. The solution of using self-report tools may not suffice, as self-reports may also be affected by social desirability and demand, and capture behavioral intentions, which are likely to be biased toward more liberal intentions relative to actual behaviors (Kollmayer et al., 2018). For example, when participants are specifically asked which toys they would prefer hypothetically (e.g., Weisgram and Bruun, 2018), they are likely to realize the goal of research and might tilt they preferences accordingly. Thus, the use of such measures limits the ability to conclude that parents are indeed acting naturally as they would in their daily lives. Such ability is critical for researchers, particularly if their goal is to be able to affect the behavior and reduce its prevalence.

The main goal of the current work is therefore to overcome these drawbacks and provide an unobtrusive, easy-to-implement, behavioral measure that will capture parents' tendency to promote the gender-typing of their children, by an actual choice of toys that vary on their gender-typicality. We relied on extensive research on the manifestations of gender parenting, that deals with parent's choices of toys for their children and developed the Gender Toy Choice (GTC) measure, in which parents are asked, via an online study, to actually choose toys that they want (and indicate which toys they do not want) to be sent as a gift for their preschool son or daughter. The use of GTC will enable more coherence in the measurement of this aspect of gendered parenting in future research, including its use in manipulation studies and causal designs, designated to attenuate gendered parenting.

This index can be meaningfully interpreted in the context of well-established constructs in the literature on gendered parenting. Specifically, a higher score on the wanted gifts index and a lower score in the unwanted gifts index (which reflects a tendency to avoid more strongly counter-stereotypic toys) indicate a tendency to reward gender-conforming behavior and discourage non-conforming behavior—processes referred to in the literature as gender reinforcement and gender policing (Fagot and Hagan, 1991; Kane, 2006). Parents who provide gender-typical toys and avoid or reject gender-atypical ones may be signaling approval of normative gender expressions while subtly discouraging behaviors that deviate from these norms. Such parental responses have been shown to shape children's understanding of acceptable gender roles, thereby reinforcing binary conceptions of gender (Endendijk et al., 2014; Blakemore and Hill, 2008). Thus, the wanted/unwanted gifts indices capture not only toy preference but also the affective and social meaning parents attach to children's gendered behaviors, reflecting mechanisms through which parents may actively reproduce gender norms.

Importantly, we wish to clarify that even though the GTC measure provides a way to assess parental behaviors that reinforce traditional binary conceptions of gender, we acknowledge that gender exists on a spectrum encompassing diverse identities and expressions (Hopkins and Richardson, 2021; Levitt et al., 2024; Monro, 2019; Morgenroth and Ryan, 2021; Salinas-Quiroz and Sweder, 2023; Wood and Eagly, 2015). Nevertheless, societal perceptions often adhere to a binary framework of men and women (Hyde, 2005; Saguy et al., 2021), and the measure we developed assesses this very adherence in the domain of parenting. Specifically, we ask parents to choose between highly gendered or less gender-typed products for their children. This methodological choice reflects the societal prevalence of binary gender views, rather than our endorsement of them. By enabling the investigation of actual parental choices, this tool may help researchers deepen the understanding of the forces and dynamics sustaining traditional gender norms and contribute efforts to promote gender diversity and equality.

## The current research

### GTC development and pre-test

We first developed and pre-tested the new GTC measure. Then we run a pilot study in Israel, including only mothers, who filled out this new measure and enabled an initial assessment of the predictions (see below). This was followed by Study 1, held also among Israeli parents, mothers and fathers, and by Study 2 [that was pre-registered (https://osf.io/259fx)] and was held among American parents, both mothers and fathers.

To explore the potential generalizability of our findings, we conducted the study in two culturally distinct yet structurally comparable settings: Israel and the United States. While both are Western societies in which gender remains a salient axis of social organization, they differ in how gender-related expectations are articulated and regulated. For instance, Israeli society is shaped by a gendered language system and a military ethos that tends to elevate stereotypically masculine values (Ben-Shalom, 2012; Sasson-Levy, 2003), whereas American discourse, especially in liberal circles, places stronger emphasis on inclusivity and gender-sensitive norms (Ridgeway, 2011). Examining parents' responses across these two contexts, allows us to explore whether gendered parenting behaviors reflect culturally specific expressions or more universal psychological mechanisms.

All studies were conducted in accordance with ethical guidelines for research with human participants and were approved by the Ethics Committee of Reichman University. Informed consent was obtained in writing as part of the online questionnaire, and participants were informed that they could withdraw from the study at any time without penalty. Participants' privacy and autonomy were fully respected throughout the process. Given that participants were unaware that their toy choice would serve as a behavioral measure of gendered parenting, they were debriefed at the end of the study and were invited to contact the researchers with any questions or concerns. The promised raffle was conducted as described, and five participants from each sample were randomly selected to receive a toy voucher.

We first comprised a set of preschool children's toys. The toys set was based on the previous research Blakemore and Centers (2005) conducted in the United-States, in which participants rated 126 toys on a nine-point scale ranging from 1 (extremely feminine) through 9 (extremely masculine) with 5 indicating “gender neutrality”. For the Israeli parents, we selected 35 toys from that list according to their gender typicality ratings (extremely feminine toys, somewhat feminine, gender neutral, somewhat masculine, and extremely masculine), and adapted some to ensure all are available in Israeli stores, all roughly cost the same (~30 USD), and all fit the 2.5–7 age range (with half fitting the younger children and half fitting the older children in this range). We then ran a pretest among Israeli adults (*N* = *82*; 59 women, *Mage* = *25.41, SD* = *2.15*; 23 men, *Mage* = *26.04, SD* = *3.05*) and obtained gender-typicality ratings for each toy, on the same nine-point scale.

Based on this pretest, we created a final set of 20 toys, organized into five categories based on their average gender-typicality ratings: highly masculine (scores between 8.0 and 9.0), somewhat masculine (6.0–7.9), gender-neutral (4.0–5.9), somewhat feminine (2.0–3.9), and highly feminine (1.0–1.9). The gender typicality scale was treated as continuous, and the category boundaries were inclusive. Within each category, two toys were selected for younger preschoolers and two for older preschoolers. Importantly, the final gender-typicality score of each toy was derived from the average rating it received in the pretest and retained as a continuous numerical value ranging from 1 (extremely feminine) to 9 (extremely masculine).

**Figure 1:**
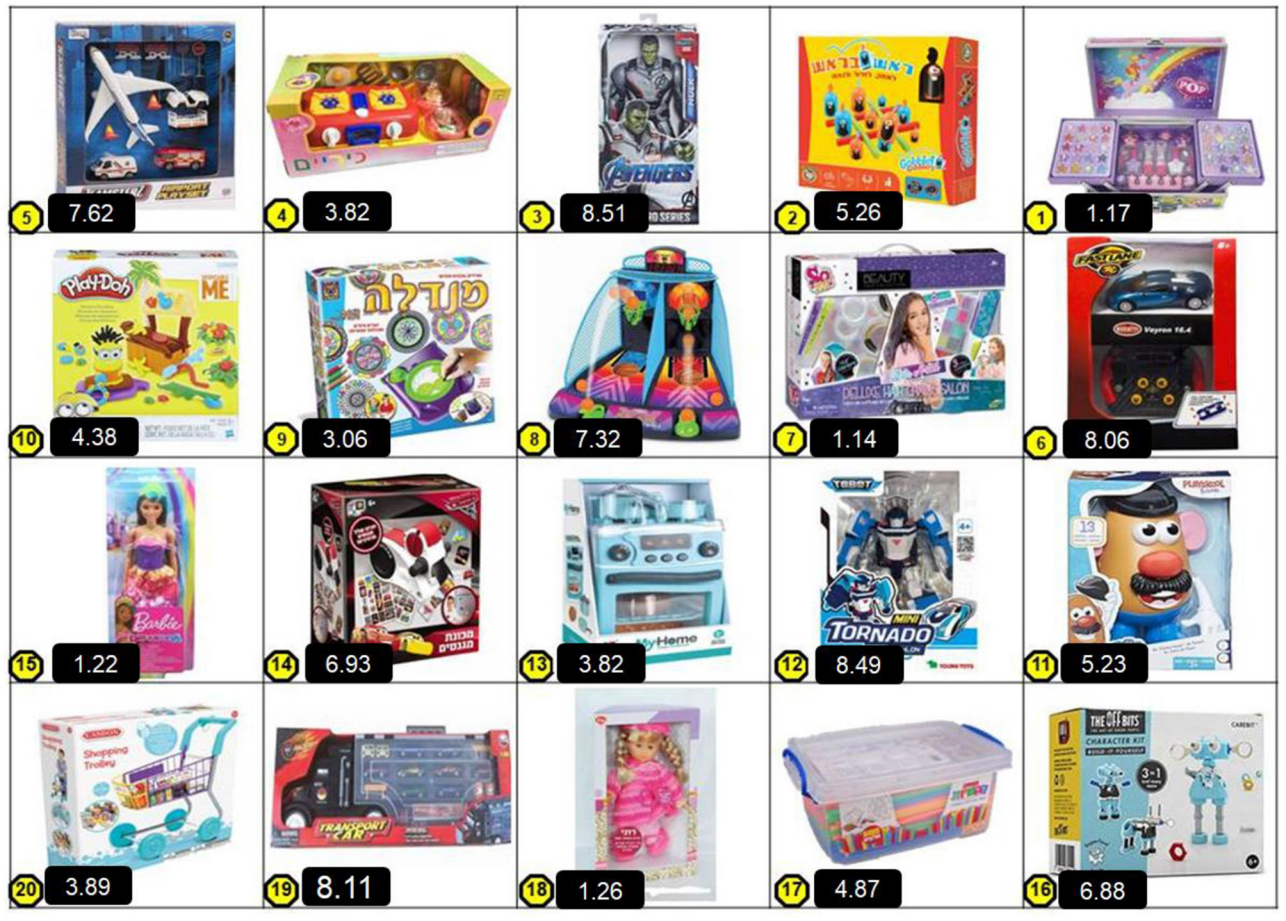
Final set of toys presented to Israeli parents, with gender-typicality scores obtained in the pretest. Scores were used for coding and constructing the indices of the GTC measure but were *not* presented to parents.

These scores were later used to compute two behavioral indices for each participant: the *wanted gift* index and the *unwanted gift* index. Each index was calculated as the mean of the gender-typicality ratings (from the pretest) of the two toys chosen in each category (i.e., most wanted and least wanted). As such, the resulting indices formed a continuous scale as well. A full list of the toys and their assigned gender-typicality scores (not shown to participants) is provided in Table 1 (for the Hebrew version) and Table 2 (for the English version used in Study 2). In the actual study, the toys were presented to participants in a matrix displaying all 20 toys in randomized order (see Figure 1).

**Table 1:** Toys presented to the Israeli parents in Study 1 and the gender-typicality score of each toy used for coding the Index (based on pretest ratings).

**Toys**	**Gender-typicality score (ranging from 1—extremely feminine; to 9—extremely masculine)**
Make up set	**1.17**
Barbi doll	**1.22**
Big pink doll	**1.26**
Hair decoration kit	**1.29**
Mandala creation kit	**3.06**
Kitchen oven	**3.77**
Stove	**3.82**
Groceries trolly	**3.89**
Play-Doh	**4.38**
Building straws	**4.87**
Mr. potato	**5.23**
Gobblet-Gobblers game	**5.26**
Offbits (robot creation kit)	**6.88**
Cars' magnet creator	**6.93**
Table basketball	**7.32**
Airport kit	**7.62**
Remote control car	**8.06**
Transport Truck (with small cars)	**8.11**
Tobot Robot	**8.49**
Avengers action figure	**8.51**

**The Gendered Toy Choice (Hebrew version; Pilot Study and Study 1)**: This table presents the type of toys presented to the parents, and the gender-typicality score of each toy (based on the pretest ratings; *N* = 82), that was used to code the Index score. In the study, the toys were presented to parents by pictures, in a mixed-order matrix.

**Table 2:** Toys presented to the parents in Study 2, and the gender-typicality score of each toy used for coding the Index (based on pretest ratings).

**Toys**	**Gender-typicality score (ranging from 1—extremely feminine; to 9—extremely masculine)**
Hair decoration kit	**1.23**
Make up set	**1.24**
Barbi doll	**1.29**
Big pink doll	**1.4**
Stove	**2.79**
Kitchen oven	**3.41**
Groceries trolly	**3.94**
Mandala creation kit	**4.46**
Building straws	**4.95**
Play-Doh	**5.09**
Gobblet-Gobblers game	**5.13**
Mr. potato	**5.41**
Cars' magnet creator	**6.39**
Table basketball	**6.47**
Offbits (robot creation kit)	**6.63**
Airport kit	**6.71**
Remote control car	**7.71**
Transport Truck (with small cars)	**7.89**
Tobot Robot	**7.91**
Avengers action figure	**8.18**

**The Gendered Toy Choice (English Version; Study 2)**: This table presents the type of toys presented to the American parents and the gender-typicality score of each toy (based on the pretest ratings; *N* = 80; and additional sample of parents which rated few more toys, *N* = 68), that was used to code the Index score. In the study, the toys were presented to parents by pictures, in a mixed-order matrix.

We further adapted the measure and created an English Version, which was validated among American parents (Study 2). We first translated the lingual parts from Hebrew into English, and adapted the toys' set to make sure each is available in on-line international toys stores (such as Amazon). Then, we run the same pretest as we did in Israel, by asking American adults to rate a set of toys varying on their gender typicality, on a 1 (extremely feminine) to 9 (extremely masculine) scale. The initial sample included 80 American parents, that rated 32 toys. However, these ratings did not yield sufficient variance, since the rating failed to include highly masculine and highly feminine ratings, for this initial set of toys. Thus, an additional sample of American parents was recruited (*N* = *68;* all recruited via Prolific), to rate 7 more toys that were highly masculine and highly feminine.

Out of the overall list of 39 toys, we eventually selected 20 toys based on their ratings, such that we had four toys in each category of gender typicality. The final set included 20 toys to let parents choose from, and their gender-typicality scores to use for coding the measure (see in Study 2). The lists of toys used in the studies and their gender-typicality scores are detailed in Table 1 (for the toys presented in the Study 1) and in Table 2 (for the toys presented in Study 2—The English version of the GTC). A higher score in the wanted gifts index, meant a more Gendered Toy Choice, for both mothers and fathers, while for the unwanted gifts index, a lower score represented a more gendered choice for both mothers and fathers, as it captured their tendency to avoid more strongly gender counter-stereotypic toys.

As for the procedure, the GTC measure is presented to participants at the outset of the survey (after consent). Parents are told that in thanks for their participation in the study, they will be entered a raffle that will allow them to win a toy prize of their choice for their child. Then, they are asked to indicate whether their child is a boy or a girl, how old is s/he, and to choose toys for their child (out of the 20 toys set offered to them). To capture both the encouragement of gender-stereotypic expressions and the discouragement of counter-stereotypic expressions with this behavioral measure, we explain to participants that we rely on the supply in various stores, and so to be sure we can provide them with a desired gift, we ask them to list two options of most preferred toys and two options of least preferred toys for their child.

Thus, gendered parenting is assessed using two index scores, referring to the two supplementing dimensions of gendered parenting (the way the parent wants his/her child to be or behave; and the way the parent thinks his/her child should not be or behave): (1) wanted gifts index, which is calculated as the mean gender typicality rating of the two preferred toys. The gender typicality scale is reversed for girls, such that for both girls and boys, a *higher* score means more gender-typed toy choice; (2) unwanted gifts index, which is calculated as the mean gender typicality rating of the two least-preferred toys. Again, the gender typicality scale is reversed for girls, such that for both girls and boys, a *lower* score means more gender-typed behavior, since this is the *least* preferred gift.

### Face and construct, concurrent, convergent and discriminant validity

To validate the GTC measure, we aimed to establish four types of validity. First, we demonstrated that parents indeed tend to choose gender-typed toys for their children, and avoid choosing for them more gender neutral, or counter-stereotypic toys. Although the selection of gender-stereotyped toys may initially appear to reflect only face validity of the GTC measure (inasmuch as the observed behavior aligns with the construct of gendered parenting), it also provides a more substantive support for the measure's validity. Parents choosing toys consistent with traditional gender roles (despite no explicit prompting), suggests that the measure is not only relevant on its face, but that it also captures real-world gendered responses. In this sense, the observed behavior reflects the very phenomenon the measure is designed to assess, offering evidence that goes beyond face validity toward demonstrating ecological and construct validity.

Second, we aimed to establish concurrent validity, by showing that groups that are supposed to differ on this measure indeed differ at the expected pattern. Next, we aimed to establish convergent validity, by demonstrating that other measures of the same phenomenon, as well as measures of close theoretical constructs, are significantly correlated with it. Finally, we aimed to demonstrate discriminant validity, by showing that measures of unrelated theoretical constructs are not associated with it, such that the correlations with GTC are weak and insignificant.

## Hypotheses and measures

Wordings of all items are included in the online Supplementary material (SM). Unless indicated otherwise, scales were rated on a 1 (not at all/strongly disagree) to 7 (very much/strongly agree), and scores were calculated as items' mean.

### Face and construct validity

Research on gendered parenting showed that parents' tendency to encourage or promote gender-stereotypic behavior of their children, and discourage non-conforming gender behavior, can often be manifested through the rating of toys they would desire for their child (e.g., Weisgram and Bruun, 2018; Kollmayer et al., 2018). Thus, we expect the GTC measure to reflect this tendency, this time with a behavioral measure, showing that parents choose toys that are masculine for their sons, and toys that are feminine for their daughters.

We predicted that both mothers and fathers would show gendered parenting on the wanted gifts index, i.e., would choose gendered-typed toys for their children (with gender-typicality score that is significantly different from the neutral score of 5). We also predicted that parents would show gendered parenting on the unwanted gifts index, i.e., would avoid choosing more gender neutral or counter-stereotypic toys for their children (with gender-typicality score that is, again, significantly different from the neutral score of 5).

### Concurrent validity

Research on gendered parenting also showed that parent's tendency to “police” gender boundaries, is sometimes moderated by the gender of the parent and of the child (Kane, 2009, 2012). For example, fathers (vs. mothers) were found to hold a more traditional attitudes toward gender compared to mothers (e.g., Blakemore and Hill, 2008) and to react more negatively toward infant boys (vs. girls), engaging in feminine-typed play (Fagot and Hagan, 1991). Consistently, other research showed that boys (vs. girls) are more subjected to gender-typing and are judged more harshly for not conforming to gender stereotypes (Campenni, 1999; Kane, 2012; Sullivan et al., 2018), particularly by their fathers (Lytton and Romney, 1991; Spivey et al., 2018; see also Endendijk et al., 2014). Parents were also found to view feminine (vs. masculine) toys and activities as more gender stereotypical—which accounted for their greater acceptance of cross-gender conduct by girls than by boys (Campenni, 1999).

Based on these findings, we predicted that fathers would show more gendered parenting compared to mothers on the wanted gifts index, which reflects the extent to which parents prefer and choose gendered-typed toys for their children. We also expected that parents to boys will show more gendered parenting compared to parents to girls on the unwanted gifts index, which reflects parents' tendency to discourage and limit their children's engagement with gender counter-stereotypical play.

### Convergent validity

#### Correlation with other gendered parenting measures

The GTC is a new, implicit behavioral measure, which is based on the parents' actual choice of toys for their children. Since there are no existing measures of this kind in literature, in order to establish convergent validity, we used the closest indices we could find for the comparison.

##### Parents response to child's gender non-conformity

We relied on the behavioral intention questionnaire described earlier (Spivey et al., 2018), asking parents how they would respond to their child's gender non-conforming behavior. This measure includes two subscales: one assesses the extent of discomfort of the parent in face of their child's behavior, and the other assesses parent's expected behavior in that situation, i.e., to what extent he or she would do or say anything to change child's behavior—which can be considered distinct forms of gendered parenting behavior.

We adapted 6 items describing gender-atypical behaviors of the child (Spivey et al., 2018), to assess parents' responses (discomfort and attempts to change) to gender-non-conforming behaviors of their children (e.g., “Playing with Barbie dolls” for a boy, “Playing sports only with boys as playmates” for a girl). For each of these behaviors, parents were asked to rate: (1) how comfortable they would be with this behavior (ranging from: 1—extremely comfortable, to 7—extremely uncomfortable); and (2) how frequently they would do or say anything to change this behavior (ranging from: 1—never, to 7—all the time). Items were averaged to create two subscales: parent's discomfort and parent's efforts to change gender-non-conforming behaviors. There were two slightly different versions of the items, one was phrased for parents to boys (α = 0.94 for both subscales) and the other for parents to girls (α = 0.95 for both subscales). Eventually, we created an index score for each of these scales: (1) Discomfort; (2) Change, considering the child's sex.

##### Pajama scenario

Based on Spivey et al. (2018), we devised an actual scenario describing a non-conforming gendered behavior of one's child, in which the child chooses a counter-stereotypical designed pajama as a birthday present. Parents were asked to report the extent to which they would encourage or discourage their child's counter-stereotypical choice, which reflects a clear example of gendered parenting in face value.

Based on Spivey et al. (2018), we created a more detailed scenario of gender non-conforming behavior of the child, to allow parents to imagine more vividly their child in the described situation. Parents read a short paragraph describing a scenario of their child choosing a counter-stereotypical designed pajama as a birthday present (cars' pajama for girls, unicorn's pajama for boys). Parents reported on 1–7 scale, the extent to which they would encourage or discourage their child's counter-stereotypical choice (single item).

##### Gendered activity choice

We also added a face-valid activity-choice measure that was developed to the purpose of the current study. We asked parents to choose (from a given list), after school activities for their child, ranging in their gender typicality (rated by a different sample of parents). Choosing leisure activities for children as a function of their congruency with gendered perceptions about what boys and girls should engage with is another expression of gendered parenting, and thus suitable for testing convergent validity of the GTC. Nonetheless, it should be noted that while the activity choice (as well as the Pajama item and the Spivey et al.'s questionnaire) are all based on a fictitious scenarios described to parents, and therefor are considered as explicit behavioral intentions measures—the GTC is implicit and measures the actual behavior of parents choosing a gift for their child, not knowing that this choice is used as a measure in the study.

This item is asking parents to rate the desirability of different after school activities for their child, ranging in their gender typicality. Parents were presented with a list of 20 summer camp activities and were asked to choose 3 activities. The activities were rated in a pretest held among different sample of parents (Hebrew Version with Israeli parents, *N* = 60; and American parents for the American Version, *N* = 80, and additional sample of American parents that rated few more activities,^1^ *N* = *68*) to assign a gender-typicality score for each, ranging from 1 (extremely feminine) to 9 (extremely masculine). The final list of activities included 4 that were rated as highly masculine (e.g., football), 4 rated as somewhat masculine (e.g., Lego), 4 rated as gender natural (e.g., capoeira), 4 rated as somewhat feminine (e.g., cooking), and 4 rated as highly feminine (e.g., ballet dancing) (See Supplementary material). The mean of the gender typicality rating was computed of the three preferred activities chosen by the parent. As in the GTC, the gender typicality scale is reversed for girls. Thus, for both girls and boys, a higher score means more gender-typed activities choice.

We expected significant, medium-large size correlations (0.25–0.5) between the GTC (both toy choice indices—wanted gifts and unwanted gifts), and the following gendered-parenting behavioral intention measures: (a) Items asking parents about their discomfort with, and attempts to change, their child's gender-non-conforming behaviors (taken from Spivey et al., 2018); (b) An item asking parents to imagine that their child asked him/her for a counter-stereotypic pajama, and to choose their response (would comply with the child's request or not); (c) An item asking parents to rate the desirability of different after school activities for their child (supposedly for a summer camp curriculum), ranging in their gender typicality.

#### Correlation with gender-related views and conservatism

Prior research documents correlations between gendered parenting behaviors and traditional gender-related views, as with a general tendency to conservatism. Thus, we added these measures to assess their correlation with our GTC measure.

##### Gender ideology

Traditional views of gender roles, often referred to as *Gender Ideology (*which can be more or less egalitarian; Davis and Greenstein, 2009; Saguy et al., 2021) were found to predict the gender typing of children by their parents (Spivey et al., 2018). For example, parents holding more traditional views of gender roles (a non-egalitarian gender ideology) were more likely to report discomfort give their child's gender non-conforming behavior (which was also greater with sons vs. daughters) (Spivey et al., 2018). These findings are also consistent with those found in research among American adults (not particularly parents), revealing that adults who hold more traditional conceptions of gender roles, were more likely to provide negative ratings to children who violated gender stereotypes (Sullivan et al., 2018).

Gender Ideology (gender-roles attitude), was assessed using 4 items taken from the International Panel Study of Parents and children (IPSPC; Thornton and Young-DeMarco, 2001; Davis and Greenstein, 2009). Parents were asked to indicate to what extent they agree with statements describing non-egalitarian gender roles, for example: “A wife should not expect her husband to help around the house after he comes home from a hard day's work”.

##### Gender essentialism

Another gender-related construct that may correspond with gendered parenting behavior, is gender essentialism. According to gender essentialist views, girls/women and boys/men are considered different “kinds” due to inherent biological differences (Dar-Nimrod and Heine, 2011; Smiler and Gelman, 2008). Parents' gender essentialism predicted their own endorsement of gender stereotypes, and their children's gender-typed preferences (Meyer and Gelman, 2016). These findings echo other research on gender essentialism, showing that it predicts endorsement of traditional gender-role division (Tinsley et al., 2015), and of gender stereotypes (Brescoll and LaFrance, 2004; Coleman and Hong, 2008; Dar-Nimrod and Heine, 2006). Indeed, because viewing men and women as different biological “kinds” can drive a rigid perception about the domains that are suitable for men vs. women (Davis and Greenstein, 2009), it can lead to behaviors that reinforce traditional gender stereotypes across various domains, including how to raise one's children.

Gender Essentialism was assessed using an adapted 15-items version of the Gender Essentialism Scale [GES; Skewes et al., 2018; α (Study 1) = 0.89]. In Study 2, we used a shortened version by choosing 7 items out of the 15 used in Study 1 (α = 0.86), for example: “Differences between men and women are primarily determined by biology”.

##### Child rearing gender ideology

Gender ideology regarding child rearing, refers to the way parents think boys and girls should be raised in light of their prospect gender roles, i.e., parents' views about the appropriateness of toys, activities, and behaviors for/of their children, depending on their gender. Based on the theoretical perspective according to which people's views of gender roles is correlated with their own gender-typed behavior (Saguy et al., 2021; see also Wada and Beagan, 2006; Stewart, 2003; Humble et al., 2008), it is likely that parents' gender ideology with respect to child rearing, will also be associated with their actual gender-typing behavior toward their children, for example, by encouraging gender-typical behaviors and discouraging behaviors that are inconsistent with gender stereotypes.

Gender-typed attitudes toward rearing girls vs. boys (i.e., child-rearing gender ideology) was assessed using an adapted 8-items version of the Child Rearing Sex-Role Attitude Scale (Burge, 1981; Freeman, 2007; Endendijk et al., 2013). We adapted the measure to make it shorter (8 items) and include an equal number of items referring to boys vs. girls [α (Study 1) = 0.81; α (Study 2) = 0.89], for example: “quite girls would have a happier life than assertive girls”; “boys who exhibit sissy behaviors will never be well adjusted”.

##### Conservatism

Previous research showed that conservative political ideology positively correlates with gender role conformity (e.g., Rosenfeld and Tomiyama, 2021). It was also found that those more conservative in ideology, were more likely to display prejudice toward gender non-conformists, i.e., those who violate conventional gender roles (such as gay men and transgender people), in part due to their greater endorsement of binary gender beliefs (Prusaczyk and Hodson, 2020). Liberals were less likely than conservatives to endorse stereotypes about gender inversion and sexual orientation, and also less likely to use gender inversion cues in their judgments (Stern et al., 2013). As for the effect of parents' political ideology on gendered parenting, a recent study provides indirect evidence for an association between conservative ideology and such parental tendency; it was found that children whose parents reported more conservative social-political views (vs. more liberal ones), held more extreme gender prototypes and endorsed more gender stereotypes (Foster-Hanson and Rhodes, 2023). Thus, parents' level of conservatism is expected to be positively correlated with the GTC measure.

We used a single item ladder asking parents to rate their level of conservatism (0 = completely liberal; 6 = very conservative). We expected significant, yet small-medium size correlations (0.15–0.25) with the following scales: (a) Gender essentialism; (b) Gender-roles attitudes; (c) Gender ideology regarding child rearing; (d) Conservatism (how conservative vs. liberal on social issues one is).

### Discriminant validity

#### Correlation with general personality constructs

We argue that gendered parenting as captured by the GTC, will not be associated with one's tendency for social desirability, since the GTC is an implicit, unobtrusive measure (the choice of wanted and unwanted toys for one's child is both actual and doesn't imply to measure anything).

We further propose that gendered parenting, as measured in this study, is not merely a reflection of broad personality traits, but rather a distinct construct tied to gender-related ideologies and broader social motivations. Thus, we do not expect that gendered parenting would be associated with personality constructs such as openness, since the tendency to promote the gender typing of one's child mostly derives from social cognition constructs related to gender, rather than a stable personality characteristic or trait.

To test this, we selected the Openness to Experience scale from the Big Five personality inventory to assess discriminant validity, as it represents a broad personality dimension that could theoretically relate to flexible thinking. Specifically, one could expect that parents who score high on Openness may be more accepting of children's counter-stereotypical behaviors, and thus exhibit lower levels of gendered parenting. Therefore, examining the relationship between Openness and our behavioral measure allowed us to test whether the GTC captures something beyond general personality dispositions. While other personality traits (e.g., Agreeableness, Conscientiousness) might also influence parenting, we aimed to balance conceptual relevance with participant burden, given the overall length of the survey.

##### Social desirability

The short form of the Marlowe-Crowne Social Desirability Scale (Reynolds, 1982; Mikolajczak et al., 2019) was used. It is composed of 12 true/false items (7 reversed-coded), for example: “I have never deliberately said something that hurt someone's feelings”. The 0 (undesirability) to 1 (desirability) scores are summed across the 12 items.

##### Openness

Parents were asked to rate the extent to which 10 statements describe them (John and Srivastava, 1999), for example: “I see myself as someone who… Is curious about many different things” (α = 0.85).

We expected non-significant, and weak correlations (0–0.15) between gendered parenting indices (wanted gifts and unwanted gifts) and the social desirability scale (a negative small correlation); and general openness.

#### Correlation with general parenthood constructs

Similarly, we also did not expect gendered parenting to relate to general parenthood characteristics, such as parental warmth and psychological control. Although there is some evidence for possible association between parenting style (such as how warm or controlling parents are toward their child), and parents' reaction to child's gender-non-conforming conduct (Alanko et al., 2008, 2011; Spivey et al., 2018), we assert that the phenomenon of gender typing in not rooted in the nature of the relationship between the parent and the child, but rather in the parent's socio-political worldviews.

##### Parental warmth

Parental warmth was assessed using 7-item warmth/support subscale of the Parenting Styles and Dimensions Questionnaire (Robinson et al., 2001; Plunkett et al., 2007). Questions included behaviors indicative of warmth, such as “I am responsive to my child's feelings and needs” (α = 0.87).

##### Parental psychological control

Parental psychological control was assessed using an adapted 6-item version of the Parental Psychological Control Scale (Barber, 1996; Padilla-Walker et al., 2021). In the original scale, children reported how true items were for their parents. Sample items included “my parent will avoid looking at me when I have disappointed her or him”. We adapted the scale for parents' self-report (α = 0.88).

We expected non-significant, and weak correlations (0–0.15) between the gendered parenting indices (wanted gifts and unwanted gifts) and the following general measures of parenthood (unrelated to gender socialization): parental warmth and parental psychological control.

## Pilot study

As a first step for validating the GTC, we presented the measure to mothers, and tested whether they would choose toys that are significantly different than the neutral choice of 5, indicating a gendered choice (establishing face validity of the measure). Next, we examined whether boys will be more subjected to gendered parenting than girls (establishing concurrent validity).

### Method

#### Participants and procedure

Israeli Jewish mothers to preschool children (age 2.5–7) were recruited by 5 research assistants who approached mothers they knew, using a “snowball” sampling. Fifty nine mothers (out of 90 recruited) met the selection criteria of having at least one preschool child. To assess gendered parenting, mothers were asked to choose gifts for their preschool child, for whom they indicated age and sex. The sample (*Mage* = *37.21, SD* = *5.34*), included 25 mothers who chose a gift for their son (*Mage* =*5.5, SD* = *0.91*), and 34 mothers who chose a gift for their daughter (*Mage* = *5.54, SD* = *0.93*).

Mothers were presented with GTC measure and chose 2 most wanted toys and 2 least wanted toys for their child.

### Results

#### Test of face and construct validity

##### Wanted gifts

For both boys and girls, mothers chose a gender-typed gift (*M* = *6.63, SD* = *1.75* for boys*; M* = *6.59, SD* = *1.56* for girls); both means were significantly different than 5, which reflects the gender-neutral choice, *t*_(24)_ = 5.94, *p* < 0.001 for boys; *t*_(33)_ = 5.94, *p* < 0.001 for girls.

##### Unwanted gifts

As for the unwanted gifts, for boys, mothers avoided more gender neutral or feminine toys, significantly different than the gender neutral choice of 5: *M* = 2.66, *SD* = 2.17, *t*_(24)_ = −5.39, *p* < 0.001. However, for girls, mothers' choice was not significantly different from the neutral score of 5 [*M* = 4.62, *SD* = 2.53, *t*_(33)_ = −0.88, *n.s*.].

It should also be noted that the correlation between the two gifts' indices was negative in sign, suggesting that the more mothers wanted gender-typed gifts, they tended to avoid counter-stereotypical gifts; the correlation, however, was small and non-significant.

#### Test of concurrent validity

**Figure 2:**
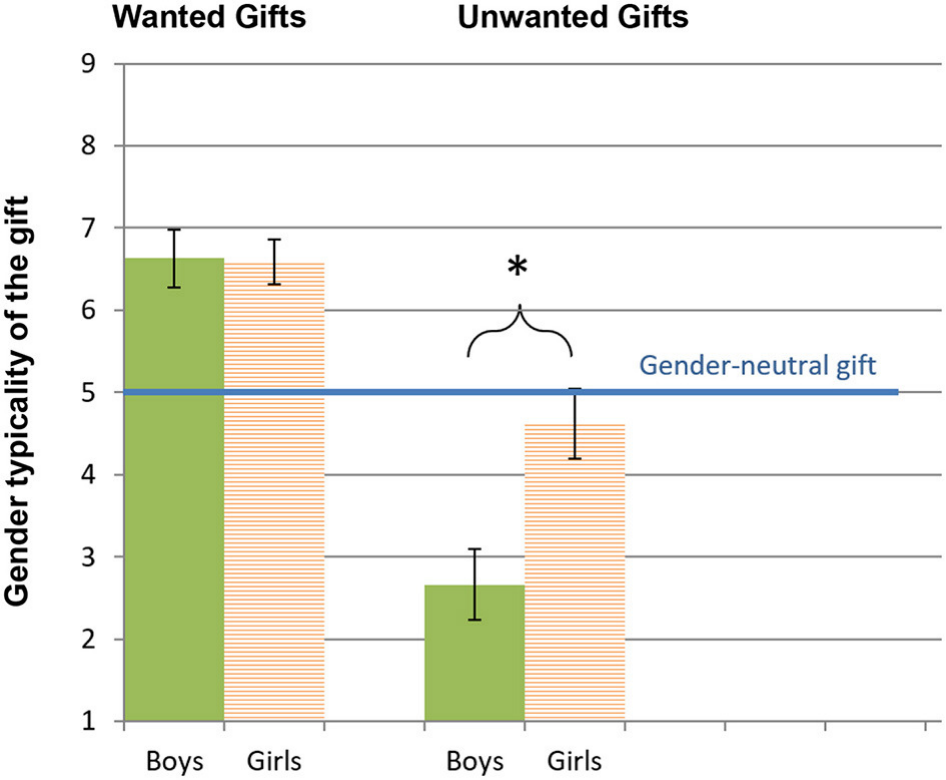
Mean and standard error of the wanted and unwanted gift indices in mothers choosing gifts for sons and daughters (Pilot Study). *Higher* wanted gift scores indicate more gendered parenting. *Lower* unwanted gift scores indicate more gendered parenting, as they reflect avoidance of gifts with low gender typicality (i.e., typical of the other gender). *p < 0.01.

Figure 2 presents the means of the wanted and unwanted gifts' indices, in mothers choosing gifts for their sons and daughters.

##### Wanted gifts

Testing for concurrent validity, child's sex did not affect mothers' scores on the wanted gifts index, *F*_(1, 57)_ = *0.01, n.s*., indicating that regardless of whether one chose a gift for son or daughter, the gift was gender-typed.

##### Unwanted gifts

For the unwanted gifts, there was a significant effect for child's sex, *F*_(1, 57)_ = *9.73, p* < *0.01, Cohen's d* = *0.82*, indicating that unwanted toys for boys were more gender-typed than for girls (i.e., mothers to sons tended more to avoid a gift that was highly feminine; *M* =*2.66, SD* = *2.17*).

Thus, in line with the literature and supporting concurrent validity of the GTC measure, results showed that boys (relative to girls) are more subjected to gender typing when proscriptive gender stereotypes are involved (Sullivan et al., 2018), as reflected in the unwanted gifts' results pattern.

## Study 1

In Study 1, we aimed to replicate the results of the pilot study, using a larger sample that includes both mothers and fathers to preschool children. This enabled to validate the GTC also among fathers, and to test whether fathers will gender type their children more than mothers (which will establish concurrent validity). In addition, Study 1 included additional measures of convergent validity: gendered-parenting behavioral intention measures (parents' response to child's gender non-conformity, pajama scenario, and gendered activity choice); and gender-related views (gender ideology (gender role attitudes), gender essentialism, gender ideology regarding child rearing and conservatism (see measures section). We expected medium-large size correlations (0.25–0.5) between the GTC and the gendered-parenting behavioral intention measures, and small-medium size correlations (0.15–0.25) with conservatism and gender-related constructs.

### Method

#### Participants

Israeli mothers and fathers (observations were independent) were recruited via an Israeli online survey company (panel4All.co.il). Out of 264 participants who received the survey link, 53 failed to complete the survey (particularly didn't indicate sex, child's sex, child's age and/or didn't choose a gift). We excluded 5 participants who indicated a child's age that was over 7 years old, and 2 who indicated child's age under 2.5 years old. This resulted in a sample of 204 parents of which 98 fathers (*M*_age_ = 38.62, *SD* = 5.00) and 106 mothers (*M*_age_ = 37.9, *SD* = 5.89). Of the fathers, 55 chose a gift for their son (*M*_age_ = 4.54, *SD* = 1.20), and 43 for their daughter (*M*_age_ = 4.53, *SD* = 1.12). Of the mothers, 57 chose a gift for their son (*M*_age_ = 4.59, *SD* = 1.33) and 49 for their daughter (*M*_*age*_ = 4.76, *SD* = 1.33).

#### Measures and procedure

As in the pilot study, participants received an online survey and were presented with the GTC measure. Then, we added measures to test convergent validity: participants completed scales assessing gender essentialism (GES; α = 0.89), gender ideology regarding child rearing (the shortened 8-items version of the CRSRAS; α = 0.81), and the Gendered Activity Choice measure (Hebrew version). Finally, they reported about demographic information.

### Results

#### Test of face and construct validity

##### Wanted gifts

On the wanted gifts index, both mothers and fathers made gender-typed choices, for both sons and daughters. For sons, the means were significantly different than the neutral choice of 5, for both mothers [*M* = *6.29, SD* = *1.50, t*_(56)_ = *6.50, p* < *0.001, Cohen's d* = *1.50*], and fathers [*M* = *6.33, SD* = *1.62, t*_(54)_ = *6.08, p* < *0.001, Cohen's d* = *1.62*]. For daughters as well, both mothers [*M* = *6.24, SD* = *1.78, t*_(48)_ = *4.87 p* < *0.001, Cohen's d* = *1.78*] and fathers [*M* = *6.38, SD* = *1.66, t*_(42)_=*5.44 p* < *0.001, Cohen's d* = *1.66*] made gender-typed choices of toys (i.e., significantly different than the neutral choice of 5).

##### Unwanted gifts

As for the unwanted gifts, replicating the Pilot Study, the means were significantly different than the neutral choice of 5, for both mothers to sons [*M* = *2.99, SD* = *2.12, t*_(56)_ = −*7.17, p* < *0.001, Cohen's d* = 2.12], and fathers to sons [*M* = *3.08, SD* = *2.00, t*_(54)_ = −*7.12, p* < *0.001, Cohen's d* = *2.00*]. However, for daughters, the means were much closer to the neutral choice of 5, such that the unwanted gifts index score did not significantly differ from the neutral choice for both mothers [*M* = *4.63, SD* = *2.20, t*_(48)_ = −*1.16, n.s*.] and fathers [*M* = *4.98, SD* = *2.42, t*_(42)_ = −*0.06, n.s*.].

In replication of the Pilot Study, the correlation between the wanted gifts index and the unwanted gifts index was significant, negative and medium size (*r* = −*0.29, p* < *0.001*), suggesting that the two GTC indices capture non-redundant, different, however complementary aspects of gendered parenting behavior (the promotion of engagement with gender stereotypic toys, vs. the limitation or prevention of engagement with counter stereotypic toys), that is consistent with the distinction between the prescriptive (what one *should* do or be) and proscriptive (what one *should not* do or be) components of gender stereotypes (Sullivan et al., 2018).

#### Test of concurrent validity

**Figure 3:**
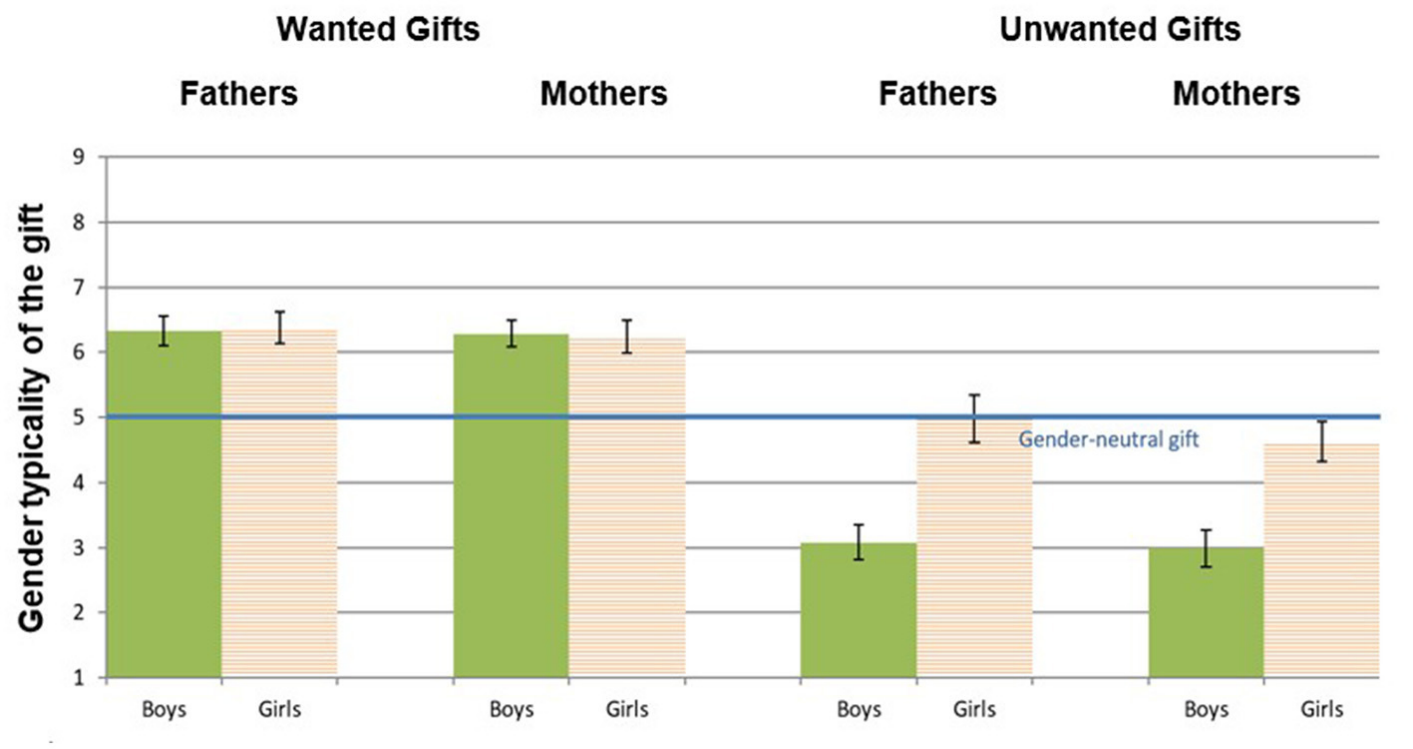
Mean and standard error of the wanted and unwanted gift indices in mothers and fathers choosing gifts for sons and daughters (Study 1). *Higher* wanted gift scores indicate more gendered parenting. *Lower* unwanted gift scores indicate more gendered parenting, as they reflect avoidance of gifts with low gender typicality.

We first conducted 2 two-way ANOVA analyses, with child's sex and parent's gender as independent variables, and gendered parenting measures (wanted gifts and unwanted gifts) as dependent variables. Figure 3 presents the means of the wanted and unwanted gifts' indices, in mothers and fathers choosing gifts for their sons and daughters.

##### Wanted gifts

On the wanted gifts index, there was no significant effect for parent's gender, meaning that fathers (*M* = *6.35, SD* = *1.63*) and mothers (*M* = *6.27, SD* = *1.63*), scored similarly on this index, i.e., they both tended to choose gender-typed toys for their children, regardless of child's sex [*F*_(1, 200)_ = *0.16, n.s*.]. There was also no significant effect for child's sex [*F*_(1, 200)_ = *0.00, n.s*.], nor interaction effect between child's sex and parent's gender [*F*_(1, 200)_ = *0.04, n.s*.].

##### Unwanted gifts

In the unwanted gifts index, replicating the Pilot Study and consistent with the predictions, child's sex, had a significant effect [*F*_(1, 200)_ = *33.5, p* < *0.001*], indicating that parents to sons tended to avoid more strongly counter-stereotypic toys (*M* = *3.03, SD* = *2.05*), compared to parents to daughters (*M* = *4.80, SD* = *2.30*). However, there was no significant effect for parent's gender [*F*_(1, 200)_ = *0.51, n.s*.], nor an interaction effect of parent's and child's sex [*F*_(1, 200)_ = *0.17, n.s*.], suggesting that mothers and fathers equally tended to avoid more gender neutral/counter-stereotypic toys for their child.

#### Test of convergent validity

Next, we examined the correlations between the GTC indices and the measures of gender essentialism and gender ideology regarding child rearing, as well as the Gendered Activity Choice measure (Hebrew version). Table 3 presents the correlations obtained and those expected. As hypothesized, the correlation with the gendered activity choice measure was positive, significant, and medium in size: *r*_(*wanted gifts*)_ = *0.42, p* < *0.01, r*_(*unwanted gifts*)_ = −*0.28, p* < *0.01*. Moreover, the correlation between the GTC measure and gender essentialism was significant for both gift indices: *r*_(*wanted gifts*)_ = *0.20, p* < *0.01, r*_(*unwanted gifts*)_ = −*0.17, p* < *0.05* (however smaller in size than predicted). The correlation with gender ideology was also significant: *r*_(*wanted gifts*)_ = *0.16, p* < *0.05, r*_(*unwanted gifts*)_ = −*0.14, p* < *0.05* (smaller in size than predicted).

**Table 3:** Summary of tested correlations in Study 1.

		**Essentialism**	**Child rearing**	**Activities**
Wanted gifts	Hypothesized			
	Observed	0.21^**^	0.16^*^	0.42^**^
Unwanted gifts	Hypothesized			
	Observed	−0.17^*^	−0.14^*^	−0.28^**^

** p < 0.01,

* p < 0.05.

**Summary of convergent validity:** This table presents a summary of hypothesized vs. observed correlations between the GTC and the other measures of convergent validity in Study 1.

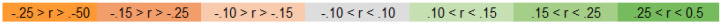

### Discussion

The pilot study and study 1 help to establish face and construct validity of the GTC measure by showing that parents (both mothers and fathers) tended to choose gender-typed gifts for their children. Unlike traditional questionnaires, the GTC presents parents with a decision-making scenario that requires a concrete behavioral choice, rather than abstract agreement with statements. This approach reveals biases that are less likely to surface in self-reports. We also established concurrent validity by showing that parents avoid more gendered gifts for their sons (vs. their daughters). Establishing convergent validity, Study 1 showed significant, small to medium size correlations between the GTC and gender ideology regarding child rearing, gender essentialism, and the gendered activity choice measure. Given the behavioral, implicit, nature of the GTC, the correlation with explicit gender views are a good indication of the measure's validity.

## Study 2

Study 2 was pre-registered (https://osf.io/3utcb/) and had two goals. First, to test for the entire set of predictions laid out in the introduction (including discriminant validity). Second, to validate the measure in an additional cultural context. In Study 2, we also added measures of personality tendencies and parenthood styles expected to not correlate with the GTC. We also added explicit measures of gendered parenting attitudes and behavioral intentions, predicted to correlate with the GTC. As in Study 1, parents first chose gifts for their child, and then answered the rest of the questionnaire items.

### Method

#### Participants

Participants were recruited via a familiar online platform (Prolific): American parents, English native speakers, having at least one child in preschool ages (2.5–7), with observations being independent. In exchange for their participation, participants were compensated as customary, and entered a raffle for a toy prize for their child (30 USD worth). Their choice of toys (2 most wanted and 2 most unwanted out of 20 toys offered) at the beginning of the survey served as an implicit measure of gendered parenting. In practice, 5 participants (randomly chosen) received an amazon gift card worth 30$.

To determine adequate sample size, we conducted an a-priori power analysis using G^*^Power. We set the following criteria: 85% power, 0.05 alpha, and a small sized correlation of 0.15—we based this estimate on our Israeli samples, showing a small-sized correlation between the gifts measure and the gender of the parent. The calculations suggested that we would need to enroll 392 participants in order to detect a small size correlation. To account for attrition and/or necessary data exclusions, we planned to collect data from more participants and stop collecting data once we have reached valid responses from 200 mothers and 200 fathers.

Four hundred and fifty three participants took the survey, of which 8 were excluded for missing values (particularly, they did not report on their sex). Two participants were excluded for failing 2 attention checks items, additional 28 for indicating a child's age that was not in requested range, and 14 more participants were excluded for missing child's age.

This resulted in a sample of 401 parents of which 200 fathers (*Mage* = *38.08, SD* = 6.72) and 201 mothers (*Mage* = *36.16, SD* = 6.63). Of the fathers, 111 chose a gift for their son (*Mage* = *4.53, SD* = *0.86*), and 89 for their daughter (*Mage* = *4.45, SD* = *0.87*). Of the mothers, 97 chose a gift for their son (*Mage* = *4.42, SD* = *0.84*) and 104 for their daughter (*Mage* = *4.44, SD* = *0.88*).

#### Measures and procedure

**Figure 4:**
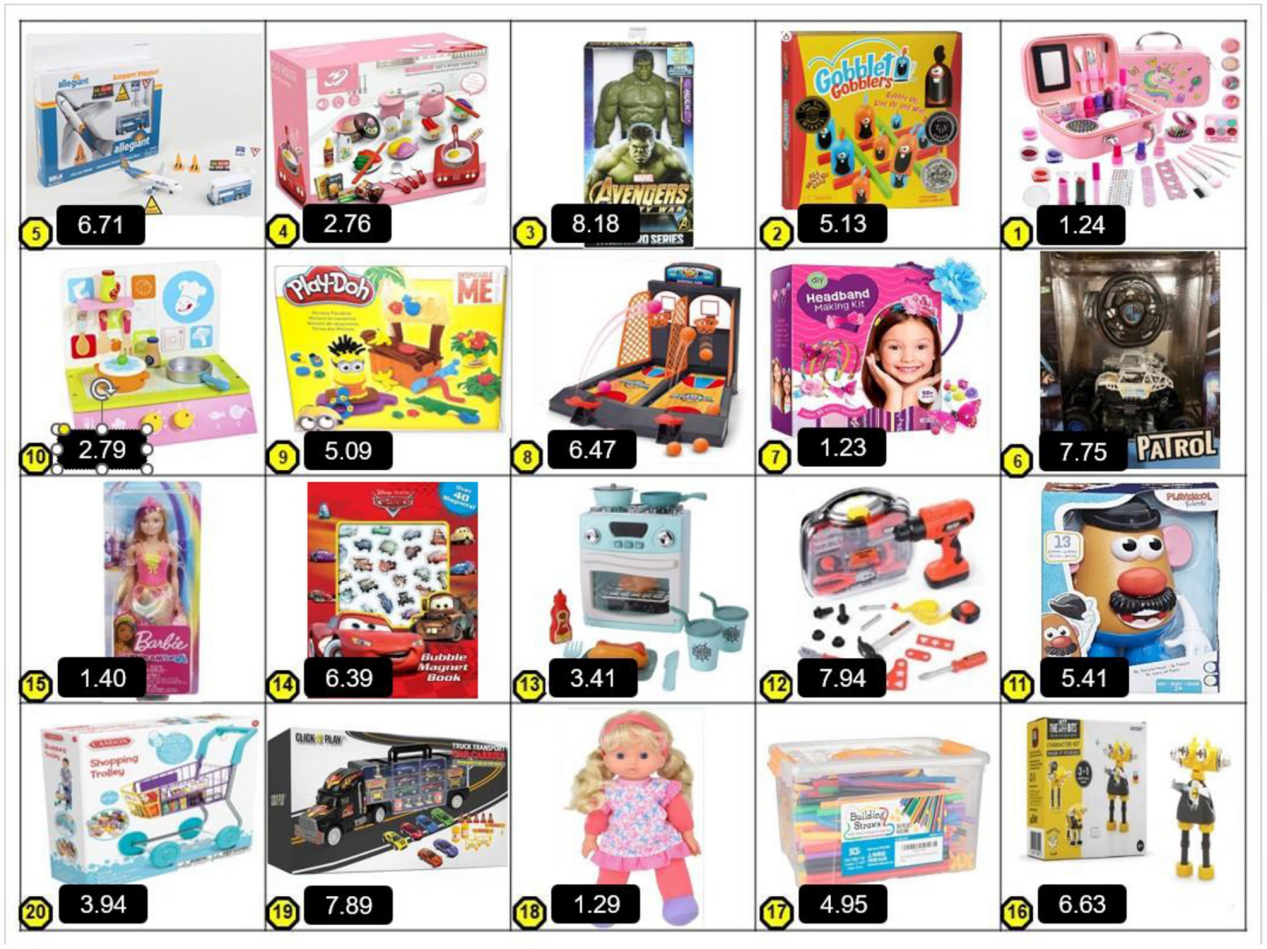
Final set of toys presented to American parents in Study 2, with gender-typicality scores obtained in the pretest. Scores were used for coding and constructing the indices of the GTC measure (English version) but were *not* presented to parents.

As in study 1, participants received an online survey and were first asked to fill in the GTC measure (English version), by choosing 2 most wanted toys and 2 most unwanted toys for their preschool child, for which they reported gender and age. Figure 4 presents the toys' choice as presented to the parents. Next, as in study 1, after choosing gifts, the parents answered the rest of questionnaire items. The scales (detailed above) were presented to the participants in an order that began with general measures unrelated to gender, then parenting item, and finally the gender-related items. The measures appeared in the following order: social desirability, openness, parental warmth, parental psychological control, gender essentialism, conservatism, gender ideology, gender ideology regarding child rearing, parents' response to child's gender non-conformity, pajama scenario, and gendered activity choice measure (English version). Participants also reported demographic information including parents' age, gender, family status, subjective socio-economic status, education level and religiosity, as well as child's sex and age.

The constructs assessed included parents' gender attitudes and behavioral intentions, some personality tendencies, and parenthood styles (see below and in SM). Finally, parents provided demographic information.

### Results

#### Test of face and construct validity

##### Wanted gifts

First, both mothers and fathers made gender-typed choices, for both sons and daughters. For sons, the means were significantly different than the neutral choice of 5, for both mothers [*M* = *6.46, SD* = *1.12, t*_(96)_ = *14.41, p* < *0.001, Cohen's d* = *1.12*], and fathers [*M* = *6.67, SD* = *1.35, t*_(110)_ = *13.01, p* < *0.001, Cohen's d* = *1.35]*. For daughters as well, both mothers [*M* = *6.38, SD* = *1.50, t*_(103)_ = *9.44, p* < *0.001, Cohen's d* = 1.50] and fathers [*M* = *6.67, SD* = *1.76, t*_(88)_ = *8.9, p* < *0.001, Cohen's d* = *1.76*] made gender-typed choices of toys (i.e., significantly different than the neutral choice of 5).

##### Unwanted gifts

Here too, both mothers and fathers made gender-typed choices, for both sons and daughters. For sons, the means were significantly different than the neutral choice of 5, for both mothers [*M* = *2.60, SD* = *1.78, t*_(96)_ = −*13.12, p* < *0.001, Cohen's d* = *1.78*] and fathers [*M* = *2.28, SD* = *1.89, t*_(109)_ = −*15.12, p* < *0.001, Cohen's d* = *1.89*]. For daughters as well, both mothers [*M* = *4.22, SD* = *1.96, t*_(102)_ = −*4.02, p* < *0.001, Cohen's d* = *1.96*] and fathers [*M* = *3.93, SD* = *1.91, t*_(88)_ = −*5.3, p* < *0.001, Cohen's d* = *1.91*] made gender-typed choices of toys (i.e., significantly different than the neutral choice of 5).

#### Test of concurrent validity

**Figure 5:**
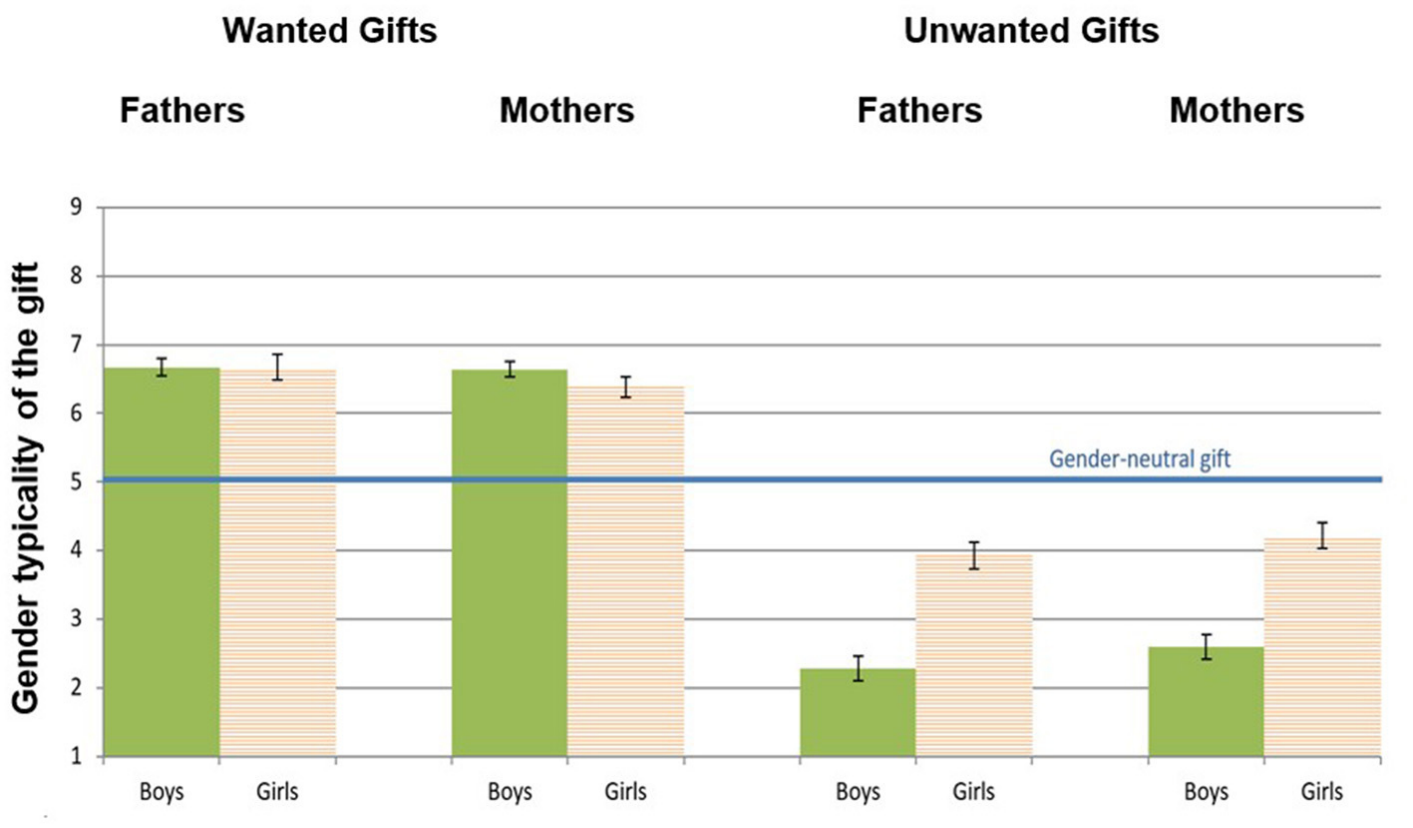
Mean and standard error of the wanted and unwanted gift indices in mothers and fathers choosing gifts for sons and daughters (Study 2).

As in study 1, to test for gender differences, we conducted 2 two-way ANOVA analyses, with child's sex and parent's gender as independent variables, and the GTC indices (wanted gifts and unwanted gifts) as dependent variables. Figure 5 presents the means of the wanted and unwanted gifts' indices, in mothers and fathers choosing gifts for their sons and daughters.

##### Wanted gifts

Replicating Study 1, fathers scored slightly higher compared to mothers, but the effect did not reach significance [*F*_(1, 397)_ = *1.20, n.s*.]. Furthermore, as in study 1, there was no effect for child's sex nor interaction between child's sex and parent's gender.

##### Unwanted gifts

Replicating the former studies, parent's gender did not yield a significant effect [*F*_(1, 395)_ = *2.66, n.s*.], nor did the interaction of parent's and child's sex [*F*_(1, 395)_ = *0.01, n.s*.]. However, as in the previous studies, child's sex had a significant effect [*F*_(1, 395)_ = *74.69, p* < *0.001*], indicating that parents tended to avoid more strongly counter-stereotypic toys for their sons (vs. daughters). This finding is in line with what we expected for establishing concurrent validity.

Thus, in both the wanted gifts and the unwanted gifts indices, parents generally tended to choose gender-typed toys for their children, establishing face and construct validity. In addition, and replicating the former studies, the unwanted gifts index showed concurrent validity by having parents to boys avoiding more strongly counter-stereotyped toys compared to parents to girls. However, we did not find significant difference between mothers and fathers in the wanted gifts index.

#### Test of convergent validity

Table 4 presents the correlations obtained and those expected. We expected medium-large size correlations (0.25–0.5) between both toy choice indices and explicit measures of gendered parenting attitudes and behavioral intentions, i.e., parents' response to child's gender non-conformity, pajama scenario, and gendered activity choice measure. As expected, all these measures, except one (the change sub-scale of parents' reactions to child's non-conforming gendered behavior), were significantly associated with both gift indices. Similarly, consistent with our predictions and replicating Study 1, both indices of the GTC were significantly correlated with gender essentialism and the gendered activity measure, with correlations ranging from small–medium to medium–large in size (some were somewhat smaller than expected; see Table 4 below).

**Table 4:** Summary of tested correlations in Study 2.

		**Discomfort**	**Change**	**Pajama**	**Activities**
Wanted gifts	Hypothesized				
	Observed	0.13^**^	0.43	0.16^**^	0.23^***^
Unwanted gifts	Hypothesized				
	Observed	−0.23^***^	−0.11^*^	−0.33^***^	−0.26^***^
		**Essentialism**	**Gender-roles**	**Child rearing**	**Conservatism**
Wanted gifts	Hypothesized				
	Observed	0.13^**^	0.05	0.03	0.09
Unwanted gifts	Hypothesized				
	Observed	−0.13^*^	−0.06	−0.05	−0.16^**^
		**Social desire**	**Openness**	**Parental warmth**	**Parental control**
Wanted gifts	Hypothesized				
	Observed	−0.04	−0.03	−0.09	−0.03
Unwanted gifts	Hypothesized				
	Observed	−0.02	0.01	0.05	0.015

*** p < 0.001,

** p < 0.01,

* p < 0.05.

**Summary of convergent and discriminant validity**: This table presents a summary of hypothesized vs. observed correlations between the GTC and the other measures of convergent and discriminant validity in Study 2.

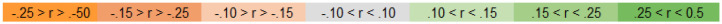

In addition, we found that the change scale (the extent to which parents would act to change their child' gender non-conformity), was significantly correlated with the unwanted gifts index, but not with the wanted gifts index. The GTC measure did not yield significant correlation with ideology measures (gender roles and child rearing gender ideology).

#### Test of discriminant validity

Finally, we examined our hypothesis predicting non-significant and weak correlations (0–0.15) between GTC and measures of personal characteristics and parenthood (social desirability, openness, parental warmth, parental psychological control). As expected, the correlations between the GTC and all these measures were small and non-significant, for both gift indices (*0.01*<*all r's* < *0.1, n.s*).

## General discussion

The main purpose of the present work was to address a gap in the literature on gendered parenting, which has limited the ability to conduct large-scale, replicable, and quantitative research using reliable measures. Our goal was to introduce a practical tool for assessing gendered parenting in the domain of child-related products, particularly toys. While gendered parenting manifests in various domains, including the encouragement of behaviors and values or product-related decisions for one's child, we chose to focus on the latter. Drawing on prior studies that utilized toy selection to assess gendered parenting, we developed an unobtrusive, easy-to-implement behavioral measure designed to capture parents' implicit tendencies to promote gender-typing of their children, by choosing for them a (more or less) gender-typed gift. The Gendered Toy Choice (GTC) measure asks parents of preschool-aged children to make an actual choice of toy (two most wanted, and two most unwanted) as a gift for their son or daughter.

The findings across studies establish face, concurrent, convergent and discriminant validity of the GTC measure, with results pattern in support of our predictions and in accordance with existing literature. First, consistent with the literature, all studies revealed that parents indeed tended to choose gender-typed toys for their children, establishing face and construct validity of this measure. Second, examining the effects of parent's gender and child's sex (to establish concurrent validity), revealed that child's sex was a significant predictor of gendered parenting, such that parents to boys tended to avoid more gender counter-stereotypic toys for their sons (i.e., scored lower in the unwanted gifts index), vs. parents to daughters.

However, this pattern did not emerge in the “wanted gifts” index, where child's sex was not a significant predictor. That is, while parents to boys were more restrictive in avoiding non-stereotypical toys, they did not differ significantly in their selection of gender-typed toys for boys vs. girls. This asymmetry between what parents actively choose and what they avoid warrants further theoretical consideration. One possible explanation for this discrepancy draws on the distinction between prescriptive and proscriptive gender stereotypes. While prescriptive stereotypes describe what children should do to align with gender norms (e.g., boys should be active, girls should be nurturing), proscriptive stereotypes emphasize what they must not do (e.g., boys must not play with dolls or show emotional vulnerability). Research has shown that violations of proscriptive stereotypes elicit particularly strong negative reactions, especially when boys engage in behaviors culturally coded as feminine (Cuddy et al., 2015; Moss-Racusin et al., 2010). Therefore, when asked which toy they would not give their child, parents may have drawn more directly on internalized proscriptive norms. This could also explain the stronger correlation between gendered ideology and responses on the “unwanted gift” index, compared to the “wanted gift” index.

Moreover, contrary to our predictions, no significant difference was found between mothers and fathers, as both mothers and fathers made equally gender-typed choices for their children. A presumable explanation for this result is that our hypothesis that fathers would enact more in gendered parenting compared to mothers was based on prior research, which used explicit, self-reports measures or obtrusive observations of gendered parenting, rather than more behavioral, implicit ones. Thus, when demand characteristics are overcome, differences between mothers and fathers might be less relevant. A support for this explanation, is found in the replicated results pattern on the child-rearing gender ideology scale: when parents were asked *explicitly*, both in Israel (Study 1) and in the U.S. (Study 2), about how boys and girls should be raised in light of gender roles, fathers (*M* = *2.83, SD* = *1.05* in Study 1; *M* = *2.88, SD* = *1.27* in Study 2) expressed significantly less egalitarian views compared to mothers [*M* = *2.51, SD* = *0.98, F*_(1, 202)_ = *5.22, p* < *0.05* in Study 1; *M* = *2.16, SD* = *1.08, F*_(1, 399)_ = *36.84, p* < *0.001* in Study 2]. This gap, however, disappears when examining behavioral tendencies to promote children's gender-typing by parents using the GTC measure, and thus showing its power and ecological validity.

As for convergence validity, results are largely consistent with predictions. The associations between the GTC and behavioral intentions measures of gendered parenting, which are self-reported (parents' response to child's gender non-conformity, pajama scenario, Study 2; and gendered activity choice measure, Studies 1–2), revealed, as expected, medium to high correlations between these scales and both wanted and unwanted indices (with the exception of “change” sub-scale yielding weak, non-significant correlation with wanted gifts index, and small significant correlation with unwanted gifts index). Given the way the GTC was presented -as a raffle-based selection for potential compensation, it is unlikely that parents recognized it as a central measure in the study. While we did not explicitly assess participants' awareness, the task was introduced at the beginning of the study, prior to any mention of gender-related constructs, and framed as unrelated to the questionnaire content. Thus, although this assumption cannot be empirically verified within the current design, the lack of shared method variance with the self-report measures strengthens the validity of the GTC as a distinct behavioral indicator.

This conclusion is corroborated by a second set of measures establishing convergent validity, including conservatism and gender related views (gender essentialism, gender ideology, gender ideology regarding child rearing). As expected, GTC was found to have significant, medium to high correlation with gender essentialism in both Studies 1–2. The correlation between GTC and conservatism (Study 2) was significant and medium in size, as expected, for the unwanted gifts index (but not for the wanted gifts index). Gender ideology regarding child rearing had significant, small-medium correlation with GTC in Study 1 but not in Study 2. The correlation with gender ideology (gender roles attitudes; Study 2) was also non-significant.

A possible explanation for the different pattern found between Study 1 and Study 2 in this regard may be rooted in cultural differences. While both societies exhibit gendered norms, the salience and acceptability of enforcing such norms may differ. Due to the strong ‘politically correct' social conventions in the U.S., American parents may have been less inclined to report less egalitarian attitudes in explicit self-report gender ideology items, due to a concern (even subconsciously), it is less socially acceptable. Future cross-cultural research should further investigate such contextual moderators.

Finally, to establish discriminant validity, we examined the associations between the GTC and theoretically unrelated constructs: parenthood scales and general personality/characteristics scales. Examining the associations between GTC and parenthood measures—parental warmth and parental psychological control (Study 2), revealed, as expected, weak, non-significant correlations between these scales and both wanted and unwanted indices. This pattern supports the discriminant validity of the GTC measure, since in shows that those are independent parenting tendencies, unrelated to gendered parenting behavior (as captured by the GTC). Similarly, correlations between GTC and personality measures examined—openness and social desirability (Study 2), also revealed, as expected, weak, non-significant correlations. Again, these results yield support of the GTC, since general personality/characteristics are shown to be unrelated to both the choice of wanted and unwanted gifts by the parents. Since social desirability may be a powerful source of influence on people's behavior, the fact that the correlation with GTC was so weak strengthen the notion that the GTC is an implicit, unobtrusive measure, that can overcome social desirability and demand problems, often characterizing existing, explicit measures of gender-typing.

As reviewed above, observational and interaction studies are complicated and expansive to perform and subjected to potential biases of the subjects and threat to ecological validity. Self-report tools, on the contrary, may also be affected by social desirability and demand, but mostly do not represent actual behavior, and are limited to capturing behavioral intentions, which are likely to be biased toward more liberal intentions relative to actual behaviors (Kollmayer et al., 2018). Thus, the usage of existing measures to assess gendered parenting behavior misses its target and limits the ability to conclude that parents are indeed acting as they would naturally do, while participating in studies. The GTC measure, being both implicit and unobtrusive, overcomes these shortcomings, while capturing two supplementing aspects of gendered parenting: the tendency to promote engagement with stereotypic toys, as well as the tendency to limit engagement with counter-stereotypic toys (or even gender neutral). The GTC thus offers a novel and potentially valuable addition to the methodological toolbox for studying gendered parenting, particularly where indirect measurement can promote our understanding of this phenomenon.

Nevertheless, this research project has few limitations. First, the Pilot Study and Study 1 were held among small sample of parents, and mostly mothers. Second, Studies 1–2 did not include the exact same set of scales, such that some of the correlations were tested only in Study 2, i.e., among American parents. Also, results did not fully replicate for measures taken in both studies (e.g., gender ideology regarding child rearing which had a medium correlation with GTC in Israeli sample, yielded non-significant correlation in American sample). More studies are needed, preferably with larger samples, to better understand the interrelations between all theoretical constructs involved.

Another limitation of this research is that it does not account for children's preferences, or, more accurately, the potential influence of parents' assumptions about their child's preferences. Thus, some parents may have chosen toys they believed their child would prefer, rather than those they themselves would endorse. Such a projection could partly confound the interpretation of the GTC score, making it less clear whether it reflects parents' gendered attitudes or their perceptions of their child's inclinations. However, if only the child's desire was playing a role in determining the choice of toys, we would not have observed the associations between GTC scores and broader ideological constructs (e.g., essentialist beliefs and SDO), as well as other gendered parenting measures assessed in Study 2 (parents' response to child's gender non-conformity, pajama scenario, and gendered activity choice measure).

Still, to better disentangle these influences, future research could include additional items assessing parents' perceptions of their child's preferences, or compare task responses with children's actual toy choices. To account for other relevant factors, future research should also incorporate additional parental variables likely influencing gendered parenting behavior, that were not accounted for in the current research (such as parents' education level, Kollmayer et al., 2018; the number and gender composition of siblings, McHale et al., 2003; Endendijk et al., 2013), to provide a more comprehensive understanding of the phenomenon.

Related to that, the list of toys we provided might include potential confounds. That is, the toys may vary in aspects other than gender typicality. It could be the case that some toys are more familiar or attractive than others, and that some toys entail more solo vs. team play (e.g., a doll vs. basketball toy, respectively). These factors may impact parents' choices, yet we do not believe that these dimensions would be predicted by ideological factors and broader motivations regarding hierarchy. In future work, the toys in the list should be matched for potential confounding factors.

Moreover, while the primary focus of the current article was on establishing the validity (different kinds of validity) of the GTC measure, we acknowledge that reliability is another essential psychometric property worthy of consideration. The decision to rely on two toy selections for both the wanted gifts index and the unwanted index was designed to balance methodological considerations: on the one hand, we aimed to maintain the ecological validity and conceal the true purpose of the measure; on the other hand, we sought to avoid relying on a single choice that might be more confounded by irrelevant factors (e.g., availability of similar toys at home). Averaging across two selections provides a more stable estimate of parental tendencies while maintaining the subtlety of the behavioral measure. The internal consistency within each index was satisfactory. In Study 1, the Pearson correlation between the two wanted toys was *r* = 0.51, *p* < 0.001, and between the two unwanted toys *r* = 0.51, *p* < 0.001. In Study 2, these correlations were even slightly higher: *r* = 0.59, *p* < 0.001 for both indices.

As for temporal stability, while the study design did not include repeated measures (i.e., we did not assess the same participants across time or different situations), the replicated patterns across independent samples offer important evidence for the measure's stability. In both studies, we found that parents tended to choose gender-typical toys for their children (choosing wanted toys significantly different from the gender-neutral choice), and avoided more strongly counter-stereotypical toys. Fathers and mothers alike displayed more Gendered Toy Choices, and the unwanted gifts index consistently showed stronger gendered choices for sons compared to daughters. These patterns were observed across two different cultural contexts (Israel and the United States), further reinforcing the measure's ability to capture a stable and meaningful behavioral expression of gendered parenting. Taken together, these findings suggest that while the GTC is a brief behavioral index, it offers a psychometrically meaningful and theoretically coherent tool for assessing gendered parenting practices in an unobtrusive manner.

Finally, the GTC was designed to fit (parents to) children aged 2.5–7, with toys that are suitable for this age group. While adapting this measure to other age groups or cultural contexts would require some time and effort, it is not overly complex. The process involves identifying a relevant list of toys, conducting a preliminary study to assess their prevalence and popularity, and running a pre-test to assign gender-typicality scores to each toy.

This research project has important implications, from methodological and practical perspectives. First, it presents a new measure for assessing gendered parenting behavior among parents to young children. Having a single, straightforward measure that is both easy to implement and analyze will promote greater consistency and coherence in the gendered parenting literature. It will also facilitate the use of larger samples and enable the replication of studies across diverse populations, in a cost-effective way. Moreover, the GTC measure has significant advantages in comparison to existing methods, by being behavioral, unobtrusive, and with high ecological validity. Finally, implementation of this new measure will offer new possibilities to study gendered parenting, and as such can contribute to applied knowledge and inform future interventions. In order to effectively assess interventions, it is highly important that a countable, valid and reliable measure of the relevant outcome (dependent variable) be put to use.

To assess the potential generalizability of our findings, we conducted the study in two distinct sociocultural contexts: Israel and the United States. While both countries are often considered Western, industrialized societies with broadly gendered social structures, they differ in meaningful ways with regard to how gender norms are expressed and negotiated. Israeli society is shaped by a gendered language system—Hebrew, in which all nouns and many grammatical forms are gender-marked, as well as by the pervasive influence of military culture, which tends to valorize traditionally masculine attributes such as strength, resilience, and leadership (Ben-Shalom, 2012; Sasson-Levy, 2003). In contrast, public discourse in the United States, particularly in more liberal segments of society, places greater emphasis on gender inclusivity and sensitivity to identity-based language (Ridgeway, 2011). These cultural differences are reflected not only in overt practices and discourse but also in the ways parents may interpret and respond to children's gendered behavior. By comparing responses across these two contexts, we aimed to explore whether the underlying psychological predictors of gendered parenting are culture-bound or reflect more universal processes.

Indeed, our previous research (Kislev et al., 2025) has shown that while explicit measures of gender ideology were lower in the U.S. sample—suggesting more egalitarian self-perceptions, behavioral measures of gendered parenting showed similar patterns to those observed in Israel. A similar pattern emerged in the current study as well, with the behavioral measures revealing comparable levels of gendered parenting across the Israeli and American samples. This highlights the value of examining how implicit biases and social norms operate across contexts that may differ in surface-level discourse, but share what seems to be a more universal underlying gender structure.

To conclude, this research project aimed to validate the new GTC measure in both Hebrew and English, in two distinct cultural contexts. The studies presented provide empirical evidence for the validity of this new measure, by supporting its hypothesized associations with other theoretical related (and unrelated) constructs. Across studies, results establish face, construct, concurrent, convergent and discriminant validity of the GTC measure, as well as high ecological validity—allowing for international usage and adaptability to different populations. The GTC carries important contribution to gendered parenting literature, as the use of GTC will enable more coherence in the measurement of this construct in future research, including in manipulation studies and causal designs, designated to attenuate gendered parenting.

## Data Availability

The datasets presented in this study can be found in online repositories. The names of the repository/repositories and accession number(s) can be found below: https://osf.io/3utcb/. In addition, Study 2 was pre-registered at OSF: https://osf.io/259fx.

## Footnotes

^1^The initial sample rated 19 activities which did not yield a sufficient range of gender typicality scores. Hence, we recruited additional sample of 68 parents which rated 5 more highly gendered activities (both highly feminine and highly masculine).
