# Supplementary material for: The Gendered Toy Choice (GTC): validating a behavioral measure of gendered parenting
Source: Front Psychol. 2025 Jul 29;16:1601339. doi: 10.3389/fpsyg.2025.1601339 (PMC12339522; doi:10.3389/fpsyg.2025.1601339)
Supplement: Supplementary file 1 [file Supplementary_file_1.docx]

**The Gendered Toy Choice (GTC):
Validating a Behavioral Measure of Gendered Parenting**

**Supplementary Materials**

**Method**

**Measures**

Since all the measures used in the Pilot Study and in Study 1, were also included in Study 2 (with additional measures), we will present below the measures of Study 2, in the order as presented to participants. The only exception is that Study 2 used the English Version of the GTC and the gendered activity choice measure.

***Social Desirability (Mikolajczak et al., 2019)***

Here are some statements that may or may not apply to you.
Please indicate the extent to which each statement describes you, by choosing True or False:

|  | **True** (1) | **False** (2) |
| --- | --- | --- |
| (1) It is sometimes hard for me to go on with my work if I am not encouraged. |  |  |
| (2) I sometimes feel resentful when I don't get my way. (R) |  |  |
| (3) There have been times when I felt like rebelling against people in authority even though I knew they were right. (R) |  |  |
| (4) No matter who I'm talking to, I'm always a good listener. |  |  |
| (5) There have been occasions when I took advantage of someone. (R) |  |  |
| (6) I'm always willing to admit it when I make a mistake. |  |  |
| (7) I sometimes try to get even rather than forgive and forget. (R) |  |  |
| (8) I am always courteous, even to people who are disagreeable. |  |  |
| (9) I have never been irked when people expressed ideas very different from my own. (R) |  |  |
| (10) There have been times when I was quite jealous of the good fortune of others. (R) |  |  |
| (11) I am sometimes irritated by people who ask favors of me. (R) |  |  |
| (12) I have never deliberately said something that hurt someone's feelings. |  |  |
| (13) We ask you to be focused while taking this survey, please choose false (*Attention check item) |  |  |

***Openness (John & Strivastava, 1999)***

Here are a number of characteristics that may or may not apply to you.
Please indicate the extent to which you agree or disagree with each statement (1- strongly disagree; 7- strongly agree):

**I see myself as someone who…**

1. Is original, comes up with new Ideas.
2. Is curious about many different things.
3. Is ingenious, a deep thinker.
4. Has an active imagination.
5. Is inventive.
6. Values artistic, aesthetic experiences.
7. Prefers work that is routine. (R)
8. Likes to reflect, play with ideas.
9. Has few artistic interests. (R)
10. Is sophisticated in art, music, or literature.

***Parental Warmth (Robinson et al., 2001; Plunkett et al., 2007)***
Items were adapted for parents' report.

**In all the following questions regarding parenting, please refer to the child for whom you chose the gift at the beginning of the survey.**Please indicate to what extant you agree with the following ideas (1- strongly disagree; 7- strongly agree):

1. I know the names of my child's friends.
2. I apologize to my child if I make a mistake in my parenting
3. I am sympathetic when my child is hurt or annoyed.
4. I praise my child when he/she is good.
5. I encourage my child to talk about his/her troubles.
6. I give comfort and understanding when my child is upset.
7. I am responsive to my child's feelings or needs.

***Parental Psychological control (Adapted from Padilla-Walker et al., 2021)***

Please indicate to what extant you agree with the following ideas (1- strongly disagree; 7- strongly agree):

1. I am always trying to change my child.
2. I want to control whatever my child does.
3. I would like to be able to tell my child what to do all the time.
4. I am less friendly with my child if he/she does not see thing my way.
5. I will avoid looking at my child when I am disappointed of him/her.
6. If my child hurt my feelings, I stop talking to him/her until he/she please me again.

***Gender Essentialism (Skewes et al., 2018)***

In Study 1 we used an adapted version of 15 item. The statements in bold were included in the **shotened version** used in Study 2.

Please indicate to what extant you agree with the following ideas (1- strongly disagree; 7- strongly agree):

1. **Differences between women and men’s personalities are in their DNA.**
2. Men and women have different abilities.
3. **Differences between men and women in behavior and personality are largely determined by genetic predisposition.**
4. Wherever you go in the world, men and women differ from one another in the same kinds of ways.
5. Women and men are fundamentally different.
6. Women are innately more nurturing than men.
7. Men and women’s personalities are more or less the same. (R)
8. Their underlying nature makes it difficult for men to learn to behave more like women.
9. **Differences between boys and girls are fixed at birth.**
10. Mothers are naturally more sensitive to a baby’s feelings than fathers are.
11. **Male and female brains probably work in very different ways.**
12. **Differences between men and women are primarily determined by biology.**
13. Women are naturally less aggressive than men.
14. **Upbringing by parents and the social environment have far greater significance for the development of sex differences than inborn differences in female and male brains** (R)
15. **People tend to be either masculine or feminine: there’s not much middle ground.**
16. **It's important that you pay attention to this study. Please tick 'Strongly disagree'** (*Attention check item)

***Conservatism***

Please indicate where it would be most accurate to place you on the following scale, in relation to your level of conservatism (0 = completely liberal; 6 = very conservative):

|  | **Level of conservatism** |
| --- | --- |

|  | 0 | 1 | 2 | 3 | 4 | 5 | 6 |
| --- | --- | --- | --- | --- | --- | --- | --- |

| Drag the cursor to the most suitable place for you: 0=completely liberal; 6=Very conservative | 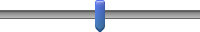 |
| --- | --- |

***Gender Ideology (4 items of the IPSPC; Davis & Greenstein, 2009)***

Please indicate to what extant you agree with the following ideas (1- strongly disagree; 7- strongly agree):

1. It is much better for everyone concerned if the man is the achiever outside the home and the woman takes care of the home and family.
2. There is some work that is men's and some that is women's, and they should not be doing each other's.
3. A wife should not expect her husband to help around the house after he comes home from a hard day's work.
4. It is more important for a wife to help her husband's career than to have one herself.

***Child Rearing Gender Ideology (adapted from Burge, 1981; Freeman***[***2007***](about:blank#ref-CR23)***; Endendijk, et al., 2013)***
 Now please relate to your attitudes regarding raising children, and indicate to what extant you agree with the following ideas (1- strongly disagree; 7- strongly agree):

1. Quiet girls will have a happier life than assertive girls.
2. Boys who exhibit sissy behaviors will never be well adjusted.
3. Girls, more than boys, should care about well-groomed appearance.
4. I feel upset when I see boys put on a dress when they play dress-up.
5. Boys, more than girls, need competitive skills.
6. It makes me uncomfortable when girls play in little league soccer.
7. Boys should be encouraged to engage in therapeutic professions
   (a nurse in a hospital, a kindergarten teacher) (R)
8. Girls who are tomboys will never be well adjusted.

***Parents response to child gender nonconformity***

We adapted 6 item describing gender-atypical behaviors of the child (Spivey et al., 2018) to assess parents’ responses to gender-nonconforming behaviors of their children. For each of these behaviors, parents are first asked how comfortable they would be with this behavior (ranging from: 1- extremely comfortable, to 7 - extremely uncomfortable); and second how frequently they would do or say anything to change this behavior (ranging from: 1- never, to 7 - all the time). Items were averaged to create two subscales: parent's discomfort and parent's efforts to change gender-nonconforming behaviors. Parents were presented with two slightly different version of the items, one for parents to boys and the other for parents to girls.

Children sometimes behave in different manners.
(a) Please indicate how **comfortable** would you be **if your son behaves in this way** (1- extremely comfortable, 7 - extremely uncomfortable):

(b) Now, assuming your son (daughter) behaves in this way, please indicate **how frequently**would you **do or say anything to change this behavior**:

Boys' version

1. Prefers girls playmates.
2. Plays with Barbie dolls.
3. Playing sports only with girls as playmates.
4. Playing female roles in make-believe play.
5. Playing 'girlish' games.
6. Dressing up as a girl / woman in dress-up games.

Girls' version

1. Prefers boys playmates.
2. Plays with soldier toys.
3. Playing sports only with boys as playmates.
4. Playing male roles in make-believe play.
5. Playing 'boyish' games.
6. Dressing up as a boy / man in dress-up games.

|  |
| --- |

***Explicit dis/encouragement of child's counter-stereotyping behavior***

A single imaginary scenario item (gendered parenting behavioral intentions in face value). Parents will choose their response to their child request to get as a present and try on a pajama in a counter-stereotypic design (different version is present to parents to boys vs. parents to girls).

The parents in your child's kindergarten have decided to buy a pajama suit for all the kids in the kindergarten. The pajama will be handed to each child on the day of their birthday celebration, in which the child will open the gift and will try on the new pajama. Parents were given a choice between these 2 pajama suits:

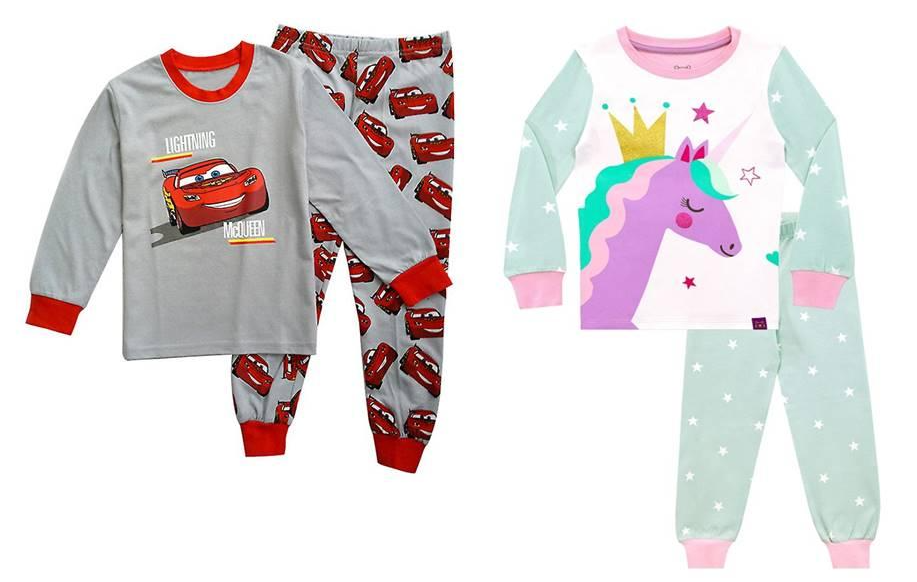


**Imagine your son (daughter) saw these options and asked you to choose**

**the unicorn (cars) pajama for his (her) birthday**. How do you think you will respond?

1. Excellent! I will praise him (her) for his (her) choice
2. That's perfectly fine with me, I'll let him (her) choose and wear it in kindergarten
3. I am willing to accept it
4. I don't mind
5. I'm not sure it's a good idea, maybe I'll be able to convince him (her) to choose the other option
6. It's not a good idea, I wouldn't want him (her) to wear it in the kindergarten
7. No way! I will explain to him (her) that I think this is inappropriate

If you like to explain your response - we would appreciate it if you could explain here: _____________________________________________________________

***The Gendered Activity Choice (Hebrew Version; Study 1)***

Table 1 presents the activities presented to the Israeli parents, and the gender-typicality score of each activity (based on the pretest ratings), that was used to code the Index score. In the study, the activities were presented to parents in a mixed-order matrix, in which they had to mark 3 boxes of their preferred activities for their child.

*Table 1*

Activities presented to the Israeli parents in Study 1, and the gender-typicality score of each activity used for coding the Index (based on pretest ratings).

| Gender-typicality score  (ranging from 1- extremely feminine;  to 9 - extremely masculine) | Activities |
| --- | --- |
| 1.85 | Dancing class |
| 2.3 | Aerobics class |
| 2.72 | Ceramics class |
| 2.92 | Paper mash class |
| 3.25 | Yoga class |
| 3.57 | Origami (paper folding) class |
| 3.67 | Cooking class |
| 3.7 | Drawing class |
| 5.47 | Math fun class |
| 5.73 | Chess class |
| 6.1 | Capoeira class |
| 6.18 | Computers class |
| 6.33 | Lego class |
| 6.53 | Judo class |
| 6.58 | Ping-pong (table-tennis) class |
| 6.7 | Basketball class |
| 6.75 | Dungeons & Dragons class |
| 6.77 | Ninja class |
| 7.9 | Soccer class |

***The Gendered Activity Choice (English Version; Study 2)***

Table 2 presents the activities presented to the American parents, and the gender-typicality score of each activity (based on the pretest ratings), that was used to code the Index score. In the study, the activities were presented to parents in a mixed-order matrix, in which they had to mark 3 boxes of their preferred activities for their child.

*Table 2*

Activities presented to the American parents in Study 2, and the gender-typicality score of each activity used for coding the Index (based on pretest ratings).

| Gender-typicality score  (ranging from 1- extremely feminine;  to 9 - extremely masculine) | Activities |
| --- | --- |
| 1.96 | Ballet dancing class |
| 2.13 | Knitting class |
| 3.12 | Yoga class |
| 3.33 | Aerobics class |
| 3.90 | Cooking class |
| 4.01 | Ceramics class |
| 4.53 | Origami (paper folding) class |
| 4.58 | Paper mash class |
| 4.79 | Drawing class |
| 5.10 | Capoeira class |
| 5.24 | Math fun class |
| 5.53 | Ping-pong (table-tennis) class |
| 5.56 | Computers class |
| 5.66 | Lego class |
| 5.70 | Soccer class |
| 5.76 | Chess class |
| 6.40 | Basketball class |
| 6.40 | Judo class |
| 6.55 | Dungeons & Dragons class |
| 7.06 | Ninja class |
| 8.26 | Football class |
